# Supplementary material for: Investigation of the Fuzzy Complex between RSV Nucleoprotein and Phosphoprotein to Optimize an Inhibition Assay by Fluorescence Polarization
Source: Int J Mol Sci. 2022 Dec 29;24(1):569. doi: 10.3390/ijms24010569 (PMC9820559; doi:10.3390/ijms24010569)
Supplement: Supplementary file 1 [file ijms-24-00569-s001.zip › ijms-2099393-supplementary.pdf]

Supplementary Information

Investigation of the fuzzy complex between RSV nucleoprotein and phosphoprotein to optimize an inhibition assay by fluorescence polarization

Silva Khodjoyan[1], Deborha Morissette[1], Fortune Hontonnou[2], Luis Checa Ruano[3], Charles-Adrien Richard[2], Olivier Sperandio[3], Jean-François Eleouët[2], Marie Galloux[2], Philippe Durand[1], Stéphanie Deville-Foillard[1], Christina Sizun[1]*

Content: Pages

NMR Spectra

Figure S1. ^{1}H NMR spectrum of **5** (699Mz, $CDCl_3$). **S2**
Figure S2. ^{13}C NMR spectrum of **5** (176 Mz, $CDCl_3$). **S3**
Figure S3. ^{11}B NMR spectrum of **5** (176 Mz, CD_2Cl_2). **S3**
Figure S4. ^{1}H NMR spectrum of **6** (699Mz, DMSO, d_6). **S4**
Figure S5. ^{13}C NMR spectrum of **6** (176 Mz, DMSO, d_6). **S4**
Figure S6. ^{11}B NMR spectrum of **6** (176 Mz, CD_2Cl_2). **S5**

UPLC Traces

Figure S7. UPLC analysis of $\mathbf{P_3}$. **S6**
Figure S8. UPLC analysis of $\mathbf{P_4}$. **S7**
Figure S9. UPLC analysis of $\mathbf{P_{5E}}$. **S8**
Figure S10. UPLC analysis of $\mathbf{P_{7E}}$. **S9**
Figure S11. UPLC analysis of $\mathbf{P_{9E}}$. **S10**
Figure S12. UPLC analysis of $\mathbf{P_{11E}}$. **S11**
Figure S13. UPLC analysis of $\mathbf{F_3}$. **S12**
Figure S14. UPLC analysis of $\mathbf{F_4}$. **S13**
Figure S15. UPLC analysis of $\mathbf{F_{5E}}$. **S14**
Figure S16. UPLC analysis of $\mathbf{F_{7E}}$. **S15**
Figure S17. UPLC analysis of $\mathbf{F_{9E}}$. **S16**
Figure S18. UPLC analysis of $\mathbf{F_{11E}}$. **S17**
Figure S19. UPLC analysis of $\mathbf{F_{11SE}}$. **S18**
Figure S20. UPLC analysis of $\mathbf{F_{11SS}}$. **S19**
Figure S21. UPLC analysis of $\mathbf{pF_{11}}$. **S20**
Figure S22. UPLC analysis of $\mathbf{BCN_{5E}}$. **S21**
Figure S23. UPLC analysis of $\mathbf{BCN_{6E}}$. **S22**
Figure S24. UPLC analysis of $\mathbf{BCN_{7E}}$. **S23**
Figure S25. UPLC analysis of $\mathbf{BCN_{8E}}$. **S24**
Figure S26. UPLC analysis of $\mathbf{BCN_{9E}}$. **S25**
Figure S27. UPLC analysis of $\mathbf{BCN_{10EE}}$. **S26**
Figure S28. UPLC analysis of $\mathbf{BCN_{11EE}}$. **S27**
Figure S29. UPLC analysis of $\mathbf{BCN_{11SE}}$. **S28**
Figure S30. UPLC analysis of **Blank.** **S29**

HPLC Traces

Figure S31. HPLC analysis of **7**. **S30**
Figure S32. HPLC analysis of 5. **S31**

UV-Visible Absorption and fluorescence Spectra

Figure S33. UV-Visible absorption spectrum and fluorescence excitation/emission spectra of $\mathbf{BCN_{10EE}}$. **S32**

NMR Spectra

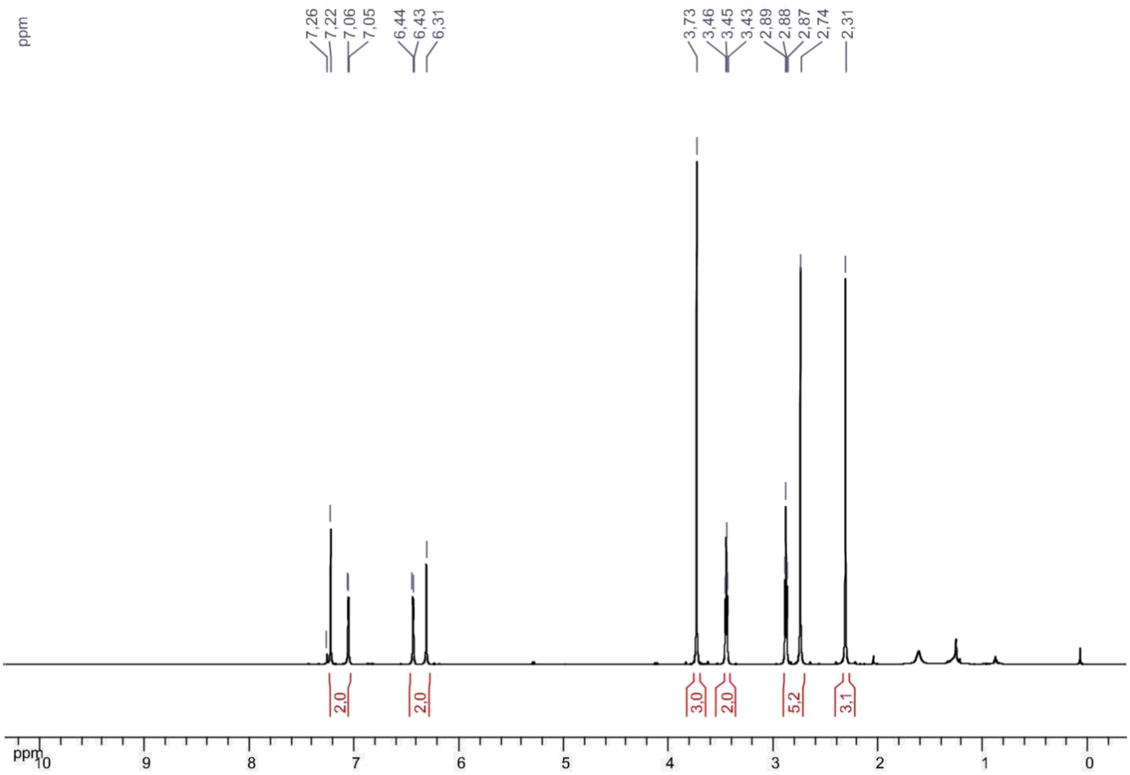

Figure S1. ^{1}H NMR spectrum of **5** (699Mz, C*D*Cl$_3$).

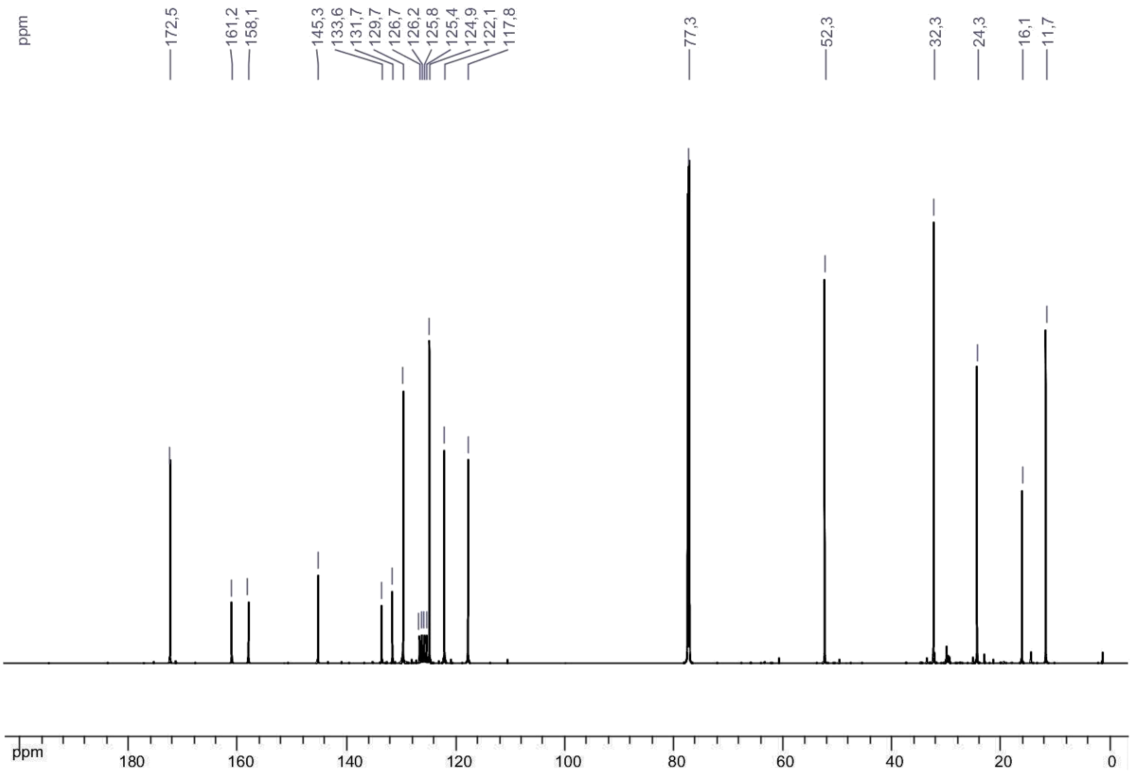

Figure S2. ^{13}C NMR spectrum of **5** (176 Mz, C*D*Cl$_3$).

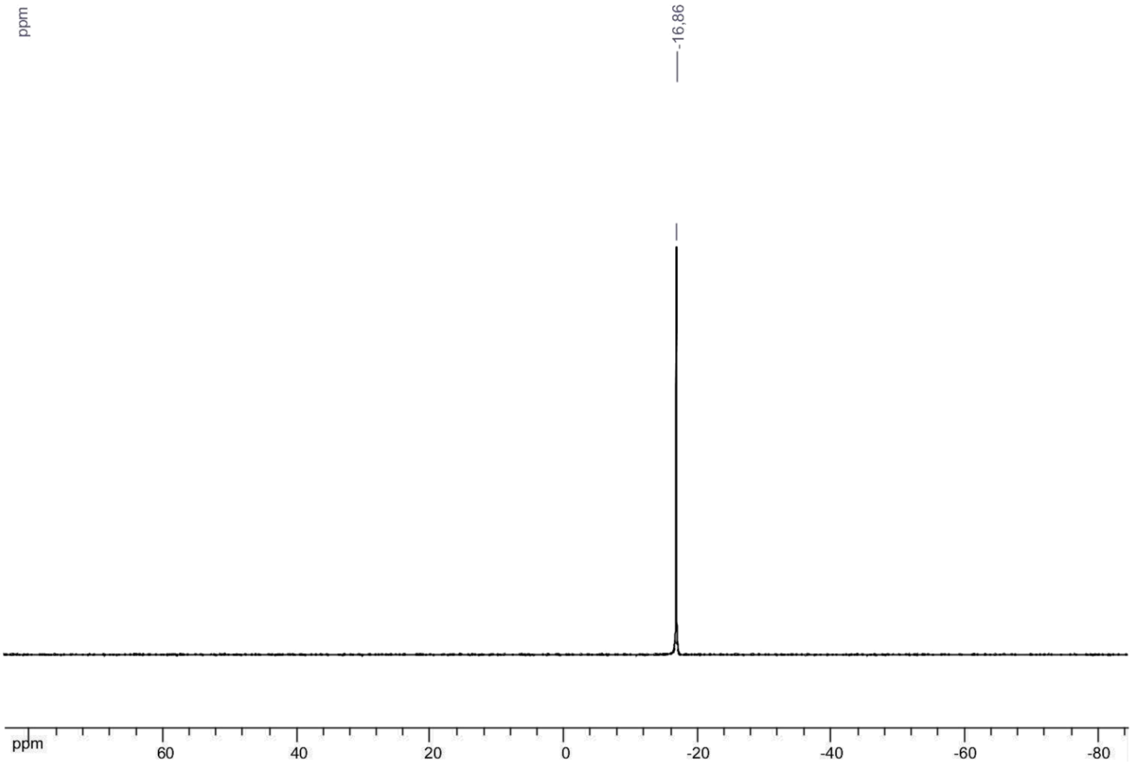

Figure S3. ^{11}B NMR spectrum of **5** (176 Mz, C*D$_2$*Cl$_2$).

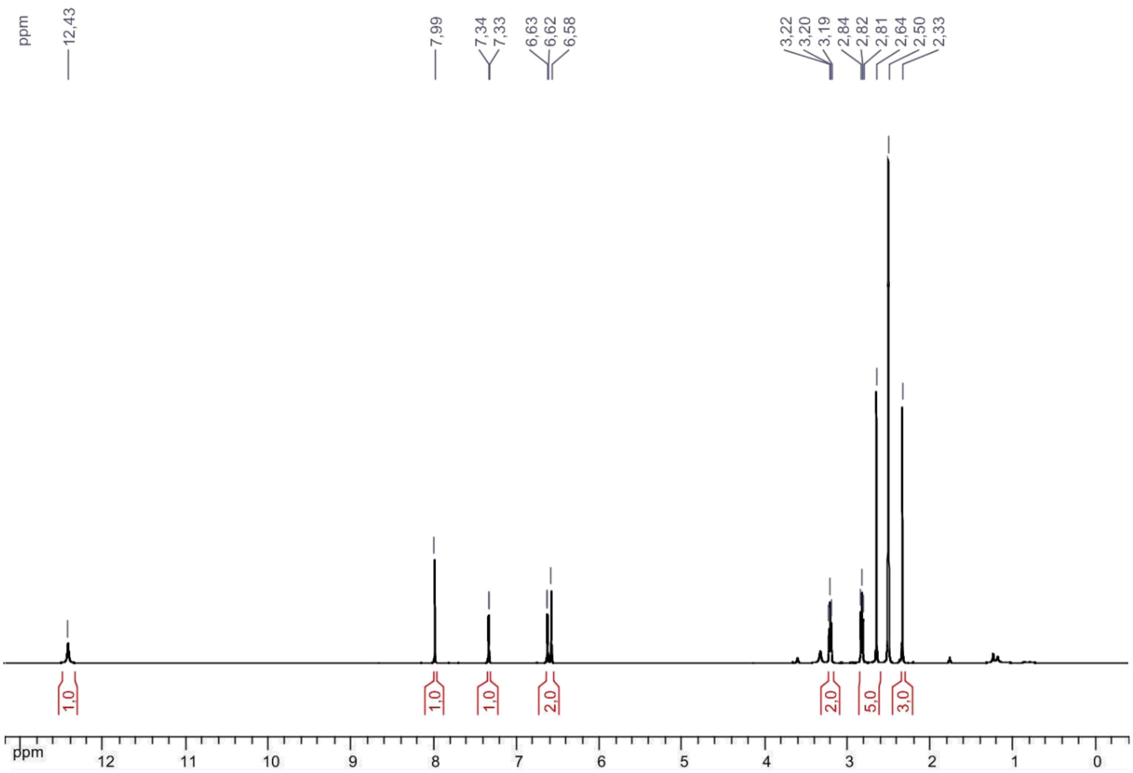

Figure S4. ^{1}H NMR spectrum of **6** (699Mz, DMSO, d_6).

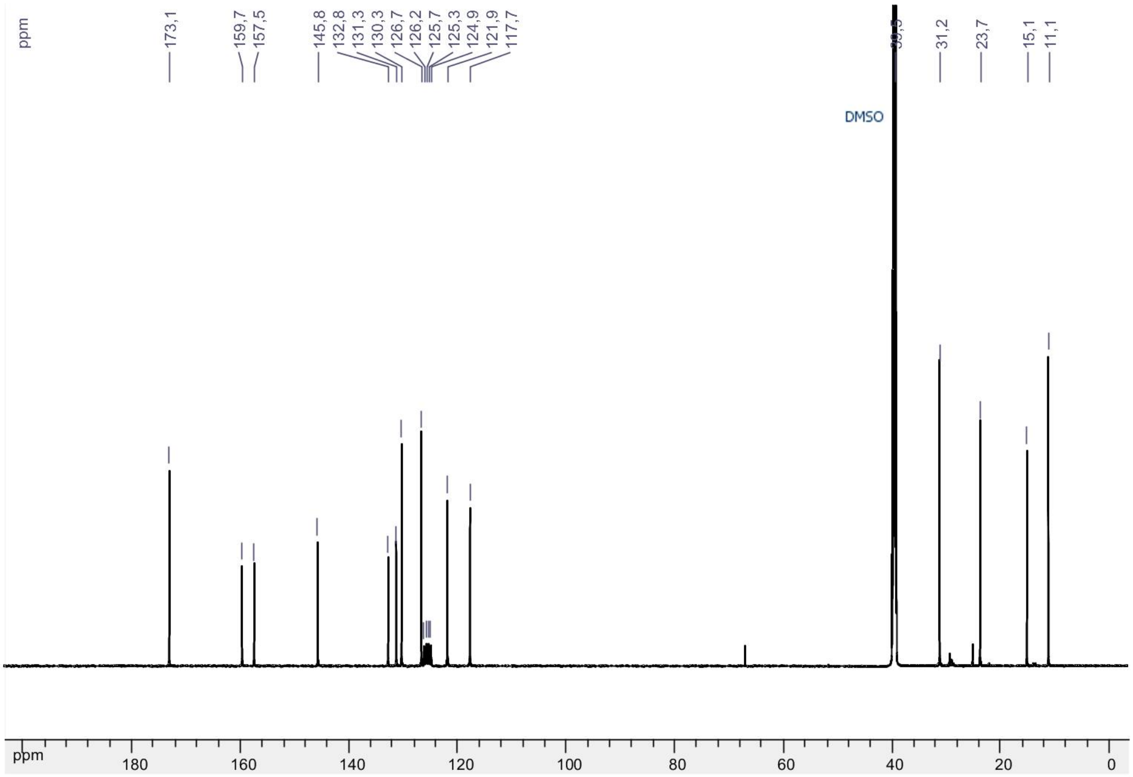

Figure S5. ^{13}C NMR spectrum of **6** (176 Mz, DMSO, d_6).

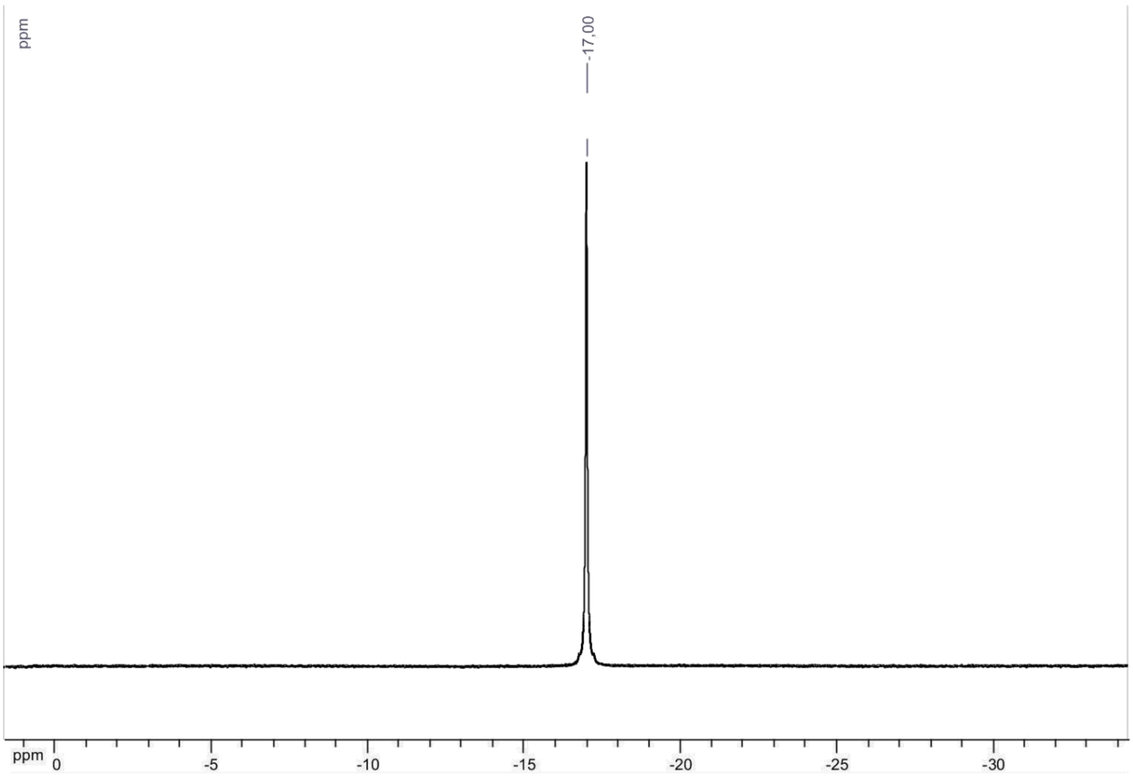

Figure S6. ^{11}B NMR spectrum of **6** (176 Mz, CD_2Cl_2).

UPLC Traces

Sample name: **P₃** | Solvent A: H_2O+0.1%HCOOH

Column: BEH C18 2.1x50 mm 1.7 µm | Solvent B: Acetonitrile +0,1% HCOOH

a)

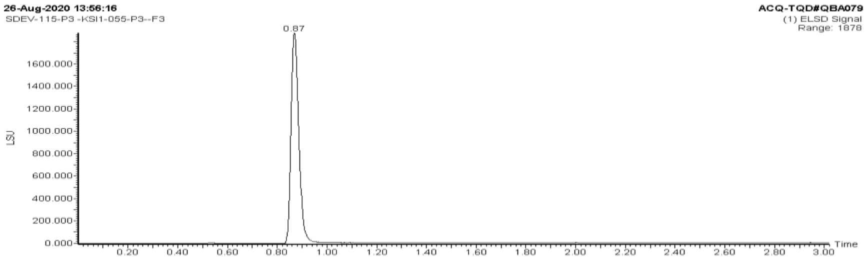

b)

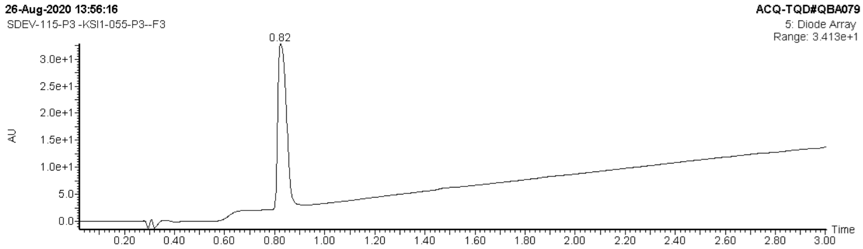

c)

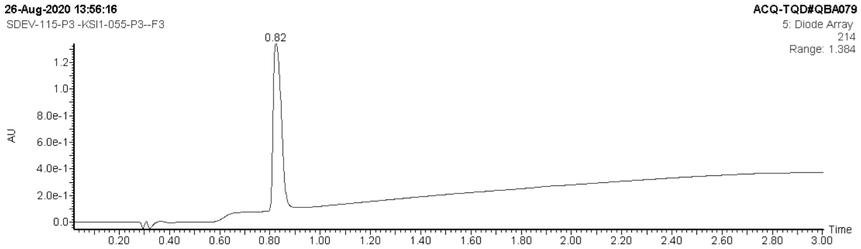

d)

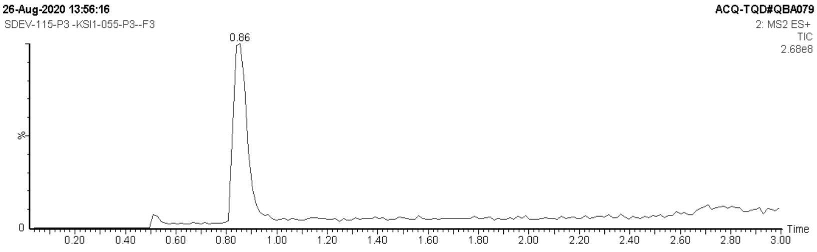

e)

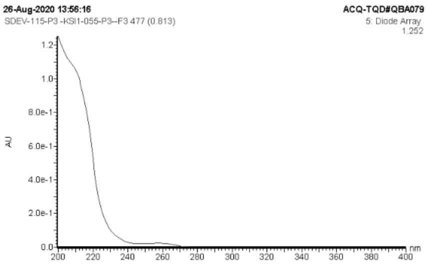

f)

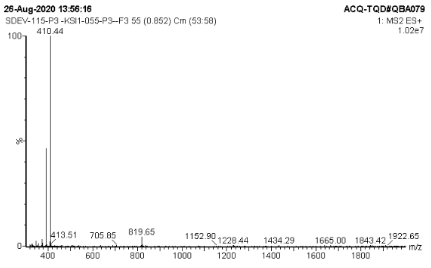

g)

Time (min)	Flow Rate (mL/min)	%A	%B	Curve
Initial	0.600	95.0	5.0	Initial
0.20	0.600	95.0	5.0	6
2.50	0.600	0.0	100.0	6
3.00	0.600	0.0	100.0	6
3.10	0.600	95.0	5.0	6
4.00	0.600	95.0	5.0	6

Figure S7. UPLC analysis of **P₃**. a) ELSD detector, b) Photodiode array detector (absorbance maximum for each compound between 220–500), c) Photodiode array detector (λ:214nm), d) Mass detector (ESI^+), e) absorption spectra of the compound, f) Mass spectra of the compound, g) gradient.

Sample name: **P4** Solvent A: H_2O+0.1%HCOOH
Column: BEH C18 2.1x50 mm 1.7 µm Solvent B: Acetonitrile +0,1% HCOOH

a)

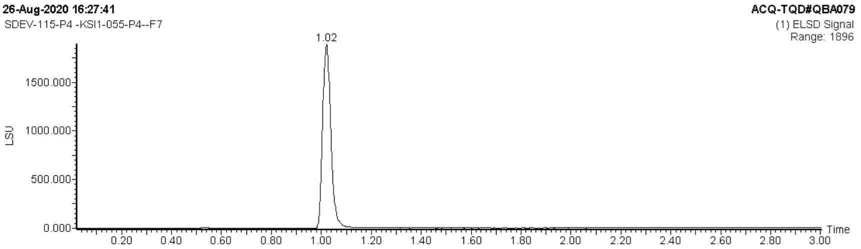

b)

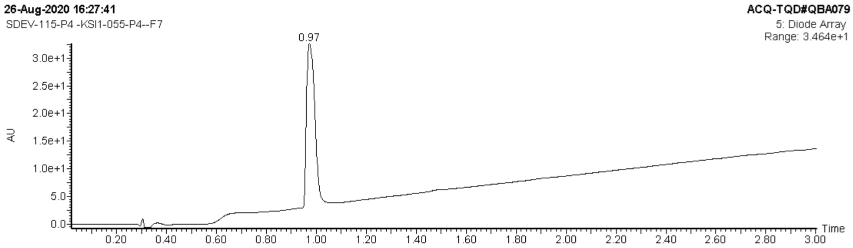

c)

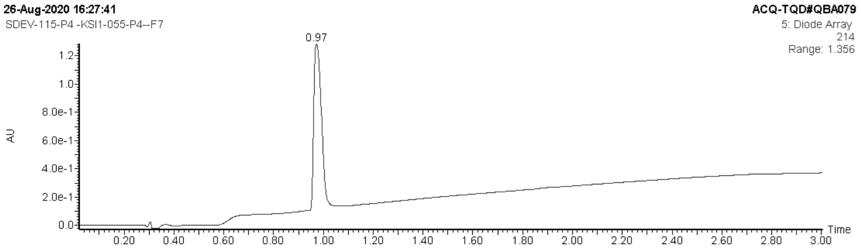

d)

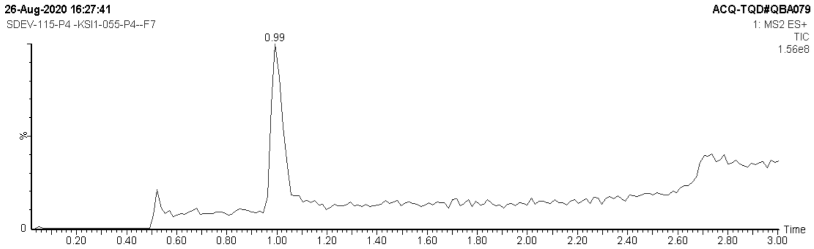

e)

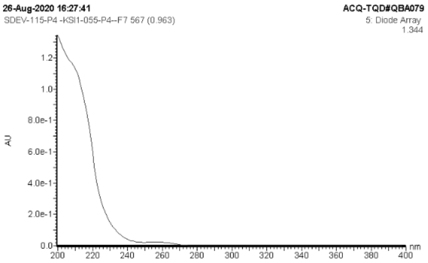

f)

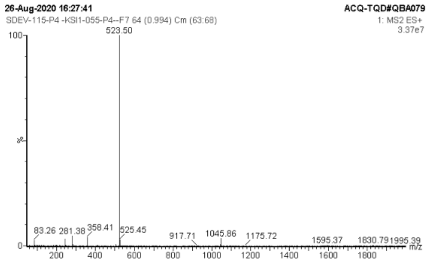

g)

Time (min)	Flow Rate (mL/min)	%A	%B	Curve
Initial	0.600	95.0	5.0	Initial
0.20	0.600	95.0	5.0	6
2.50	0.600	0.0	100.0	6
3.00	0.600	0.0	100.0	6
3.10	0.600	95.0	5.0	6
4.00	0.600	95.0	5.0	6

Figure S8. UPLC analysis of **P4**. a) ELSD detector, b) Photodiode array detector (absorbance maximum for each compound between 220–500), c) Photodiode array detector (λ:214nm), d) Mass detector (ESI^+), e) absorption spectra of the compound, f) Mass spectra of the compound, g) gradient.

Sample name: **P_{5E}**
Column: BEH C18 2.1x50 mm 1.7 µm

Solvent A: H_2O+0.1%HCOOH
Solvent B: Acetonitrile +0,1% HCOOH

a)

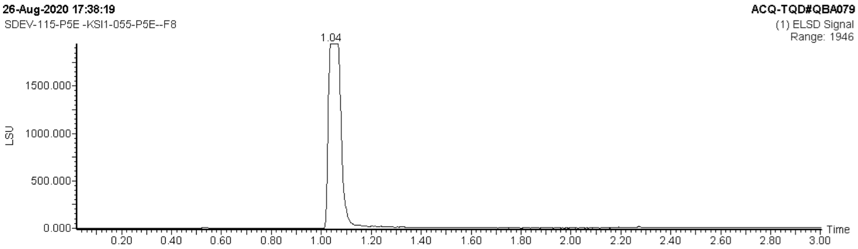

b)

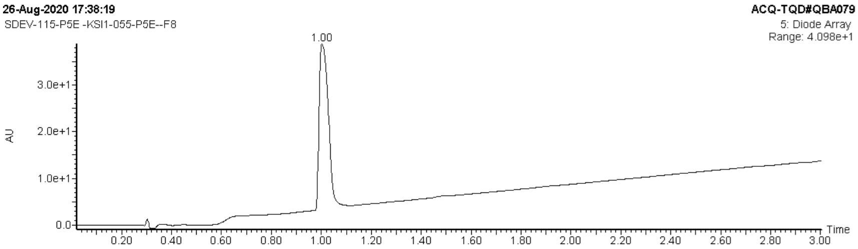

c)

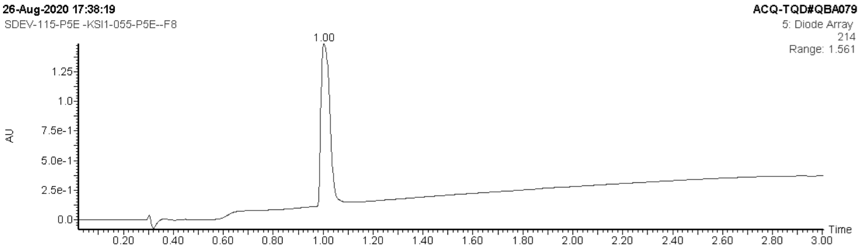

d)

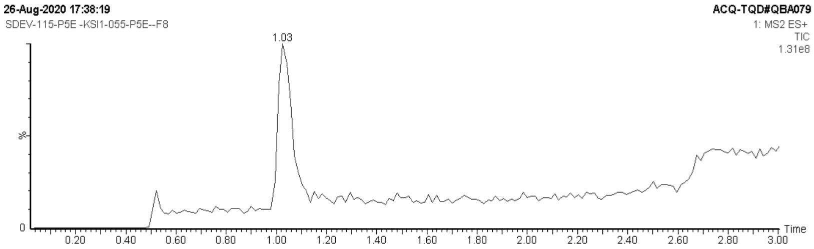

e)

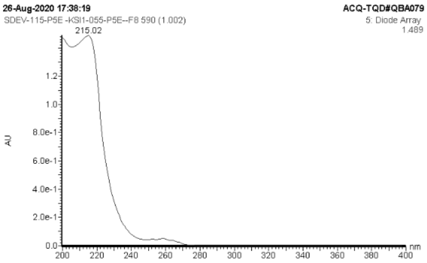

f)

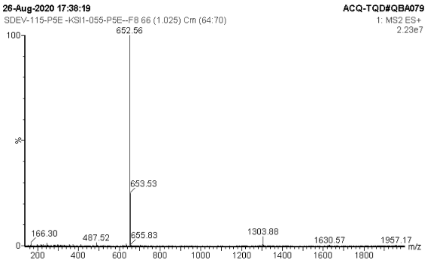

g)

Time (min)	Flow Rate (mL/min)	%A	%B	Curve
Initial	0.600	95.0	5.0	Initial
0.20	0.600	95.0	5.0	6
2.50	0.600	0.0	100.0	6
3.00	0.600	0.0	100.0	6
3.10	0.600	95.0	5.0	6
4.00	0.600	95.0	5.0	6

Figure S9. UPLC analysis of **P_{5E}**. a) ELSD detector, b) Photodiode array detector (absorbance maximum for each compound between 220–500), c) Photodiode array detector (λ:214nm), d) Mass detector (ESI^+), e) absorption spectra of the compound, f) Mass spectra of the compound, g) gradient.

Sample name: **P_{7E}**
Column: BEH C18 2.1x50 mm 1.7 µm

Solvent A: H_2O+0.1%HCOOH
Solvent B: Acetonitrile +0,1% HCOOH

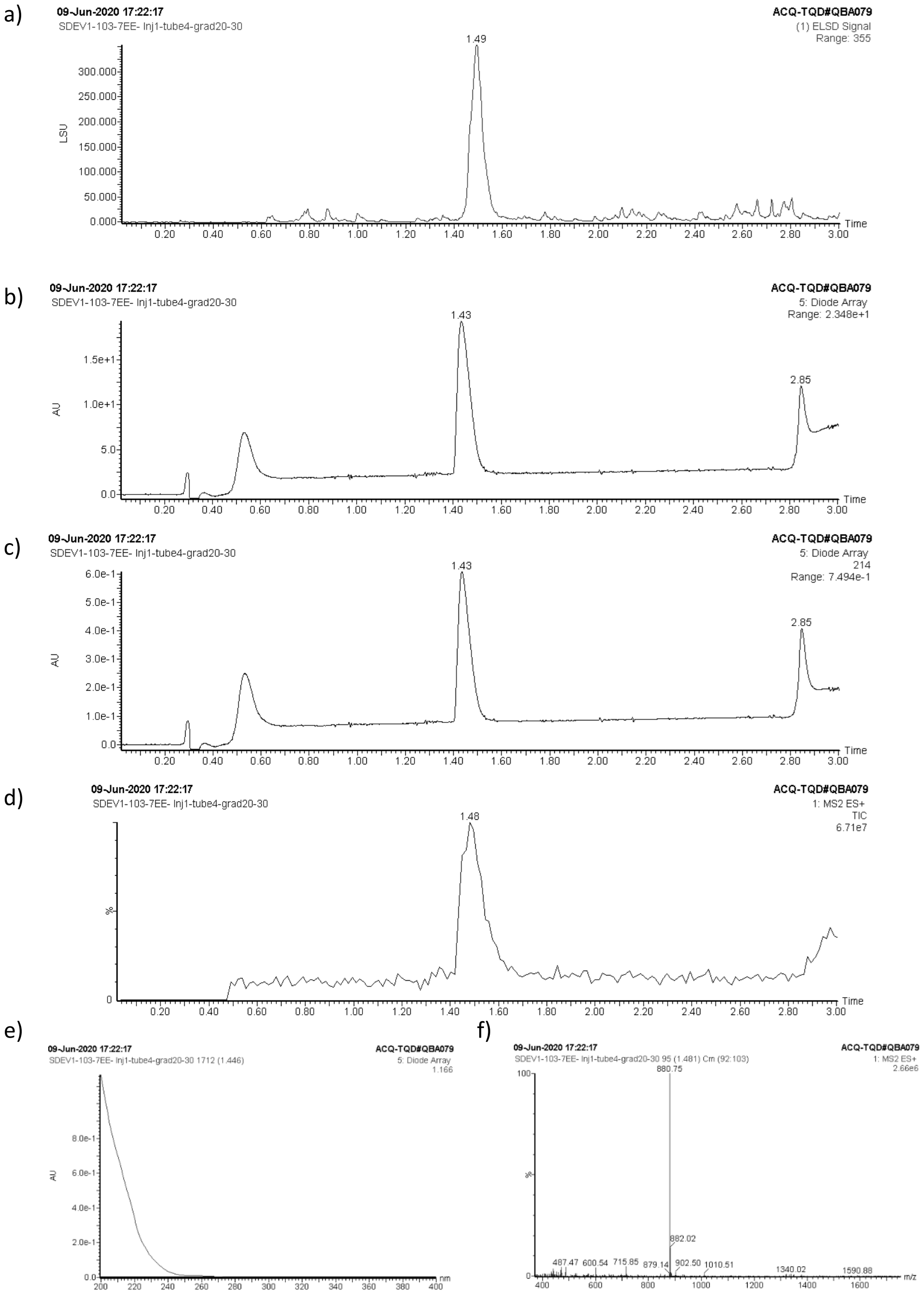

g)

Time (min)	Flow Rate (mL/min)	%A	%B	Curve
Initial	0.600	95.0	5.0	Initial
0.10	0.600	95.0	5.0	6
0.20	0.600	80.0	20.0	6
2.50	0.600	70.0	30.0	6
2.55	0.600	0.0	100.0	6
3.0	0.600	0.0	100.0	6
3.10	0.600	95.0	5.0	6
4.00	0.600	95.0	5.0	6

Figure S10. UPLC analysis of **P_{7E}**. a) ELSD detector, b) Photodiode array detector (absorbance maximum for each compound between 220–500), c) Photodiode array detector (λ:214nm), d) Mass detector (ESI$^+$), e) absorption spectra of the compound, f) Mass spectra of the compound, g) gradient.

Sample name: **P$_{7E}$** Solvent A: H_2O+0.1%HCOOH

Column: BEH C18 2.1x50 mm 1.7 µm Solvent B: Acetonitrile +0,1% HCOOH

a)

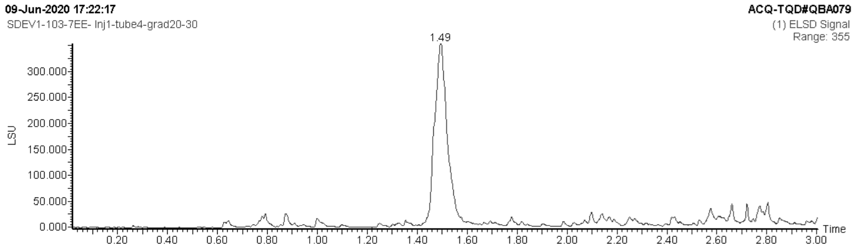

b)

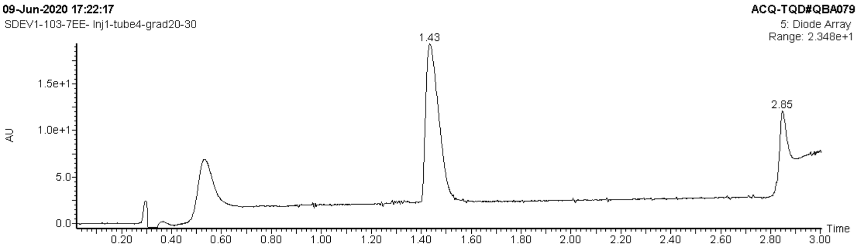

c)

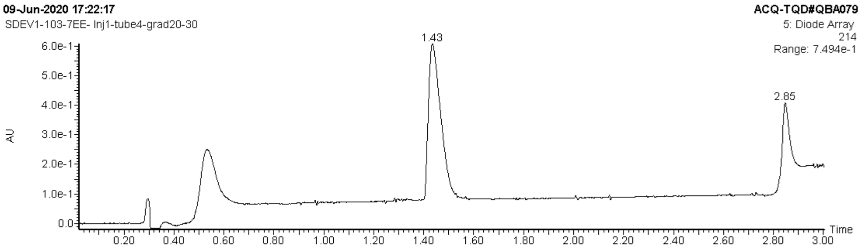

d)

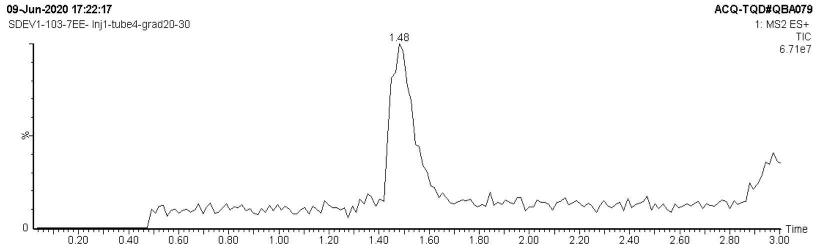

e)

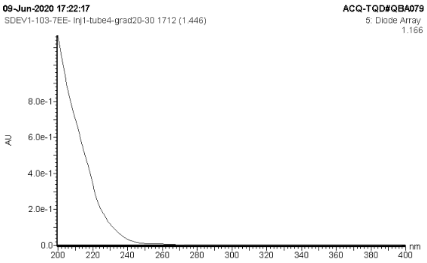

f)

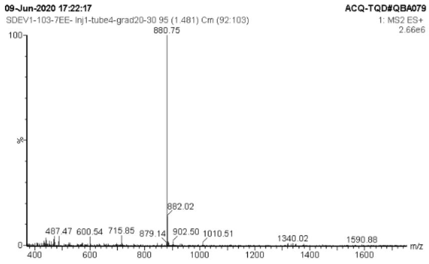

g)

Time (min)	Flow Rate (mL/min)	%A	%B	Curve
Initial	0.600	95.0	5.0	Initial
0.10	0.600	95.0	5.0	6
0.20	0.600	80.0	20.0	6
2.50	0.600	70.0	30.0	6
2.55	0.600	0.0	100.0	6
3.0	0.600	0.0	100.0	6
3.10	0.600	95.0	5.0	6
4.00	0.600	95.0	5.0	6

Figure S11. UPLC analysis of **P$_{9E}$**. a) ELSD detector, b) Photodiode array detector (absorbance maximum for each compound between 220–500), c) Photodiode array detector (λ:214nm), d) Mass detector (ESI$^+$), e) absorption spectra of the compound, f) Mass spectra of the compound, g) gradient.

Sample name: **P_{11E}** Solvent A: H_2O+0.1%HCOOH
Column: BEH C18 2.1x50 mm 1.7 µm Solvent B: Acetonitrile +0,1% HCOOH

a)

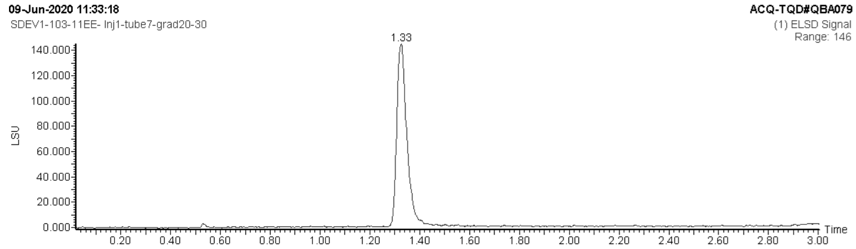

b)

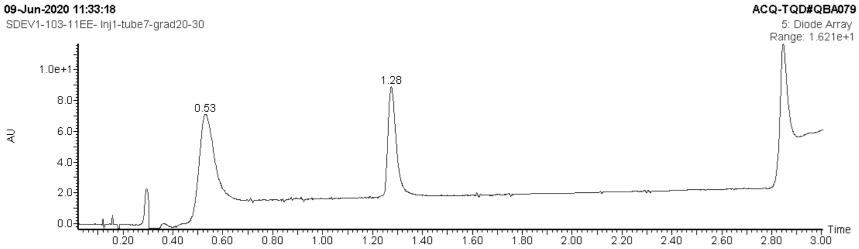

c)

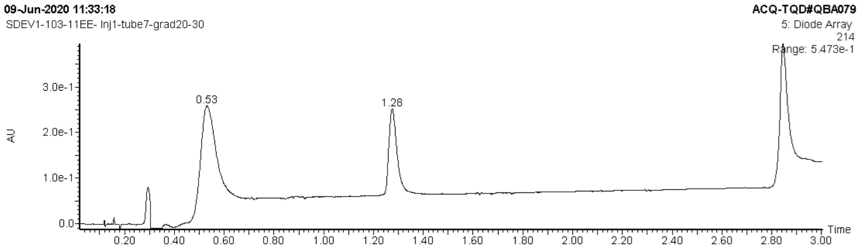

d)

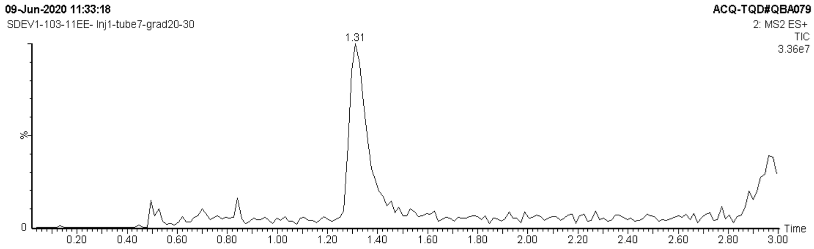

e)

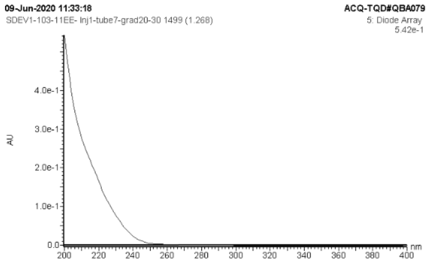

f)

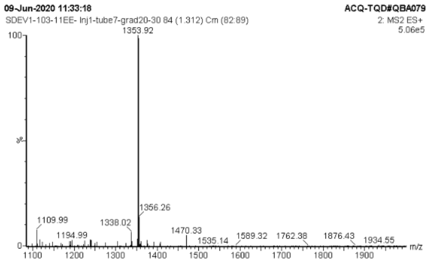

g)

Time (min)	Flow Rate (mL/min)	%A	%B	Curve
Initial	0.600	95.0	5.0	Initial
0.10	0.600	95.0	5.0	6
0.20	0.600	80.0	20.0	6
2.50	0.600	70.0	30.0	6
2.55	0.600	0.0	100.0	6
3.0	0.600	0.0	100.0	6
3.10	0.600	95.0	5.0	6
4.00	0.600	95.0	5.0	6

Figure S12. UPLC analysis of **P_{11E}**. a) ELSD detector, b) Photodiode array detector (absorbance maximum for each compound between 220–500), c) Photodiode array detector (λ:214nm), d) Mass detector (ESI^+), e) absorption spectra of the compound, f) Mass spectra of the compound, g) gradient.

Sample name: **F$_3$**
Column: BEH C18 2.1x50 mm 1.7 µm
Solvent A: H$_2$O+0.1%HCOOH
Solvent B: Acetonitrile +0,1% HCOOH

a)

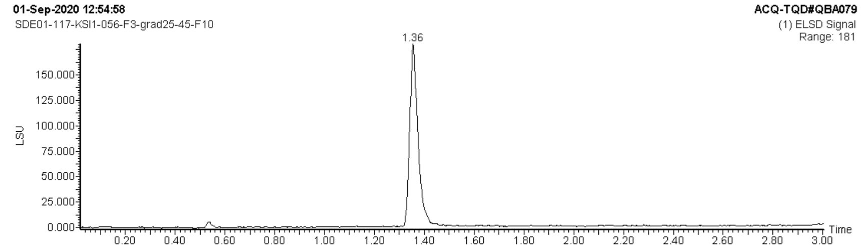

b)

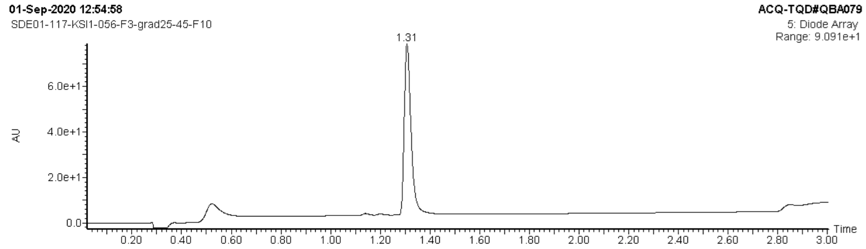

c)

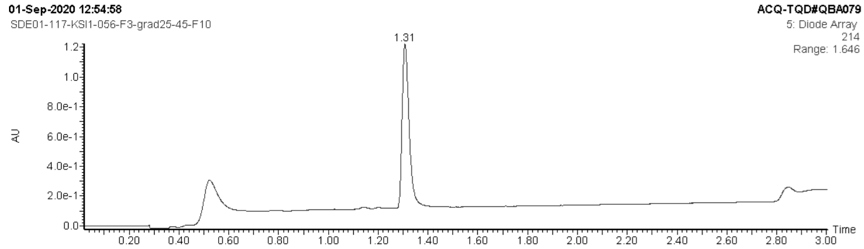

d)

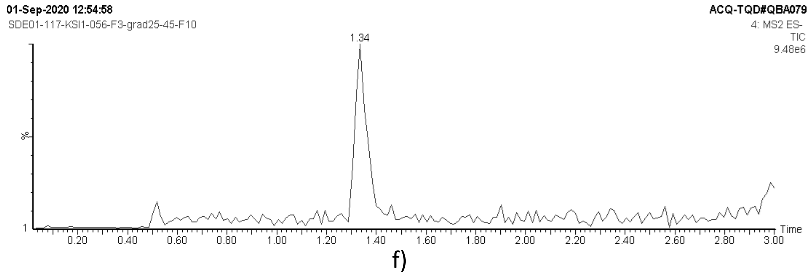

e)

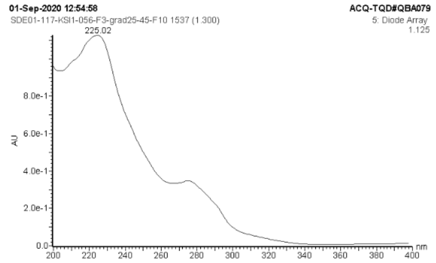

f)

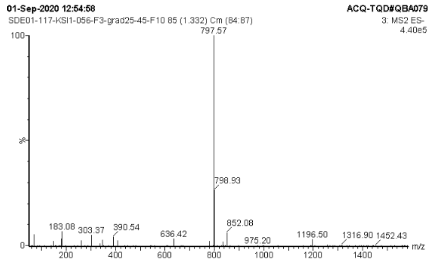

g)

Time (min)	Flow Rate (mL/min)	%A	%B	Curve
Initial	0.600	95.0	5.0	Initial
0.10	0.600	95.0	5.0	6
0.20	0.600	75.0	25.0	6
2.50	0.600	55.0	45.0	6
2.55	0.600	0.0	100.0	6
3.0	0.600	0.0	100.0	6
3.10	0.600	95.0	5.0	6
4.00	0.600	95.0	5.0	6

Figure S13. UPLC analysis of **F$_3$**. a) ELSD detector, b) Photodiode array detector (absorbance maximum for each compound between 220–500), c) Photodiode array detector (λ:214nm), d) Mass detector (ESI$^-$), e) absorption spectra of the compound, f) Mass spectra of the compound, g) gradient.

Sample name: **F4** Solvent A: H_2O+0.1%HCOOH
Column: BEH C18 2.1x50 mm 1.7 µm Solvent B: Acetonitrile +0,1% HCOOH

a)

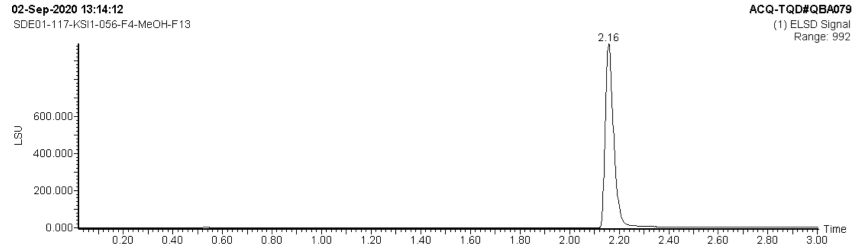

b)

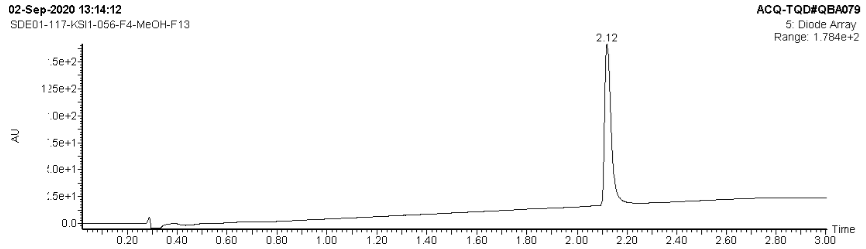

c)

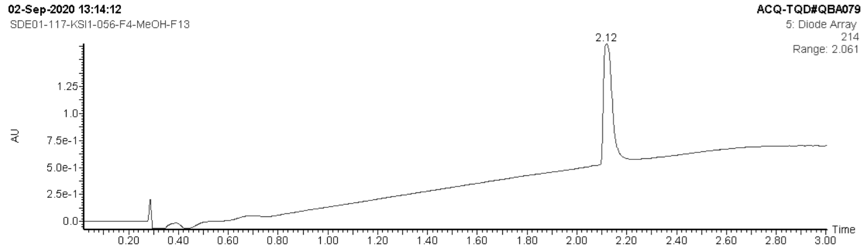

d)

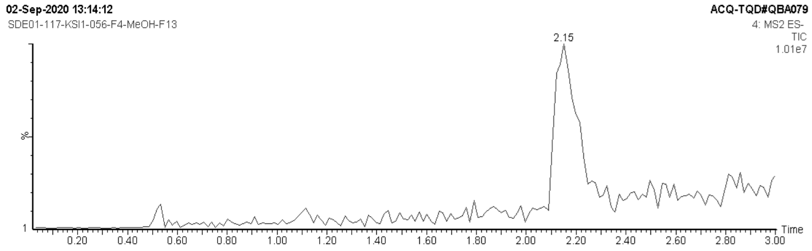

e)

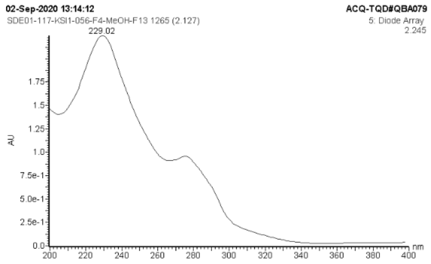

f)

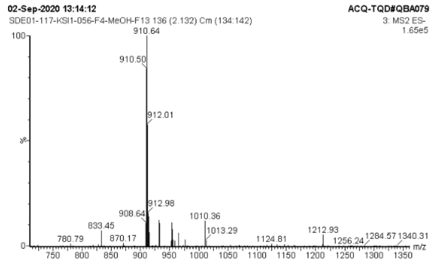

g)

Time (min)	Flow Rate (mL/min)	%A	%B	Curve
Initial	0.600	95.0	5.0	Initial
0.20	0.600	95.0	5.0	6
2.50	0.600	0.0	100.0	6
3.00	0.600	0.0	100.0	6
3.10	0.600	95.0	5.0	6
4.00	0.600	95.0	5.0	6

Figure S14. UPLC analysis of **F4**. a) ELSD detector, b) Photodiode array detector (absorbance maximum for each compound between 220–500), c) Photodiode array detector (λ:214nm), d) Mass detector (ESI$^-$), e) absorption spectra of the compound, f) Mass spectra of the compound, g) gradient.

Sample name: **F_{5E}**
Column: BEH C18 2.1x50 mm 1.7 µm

Solvent A: H_2O+0.1%HCOOH
Solvent B: Acetonitrile +0,1% HCOOH

a)

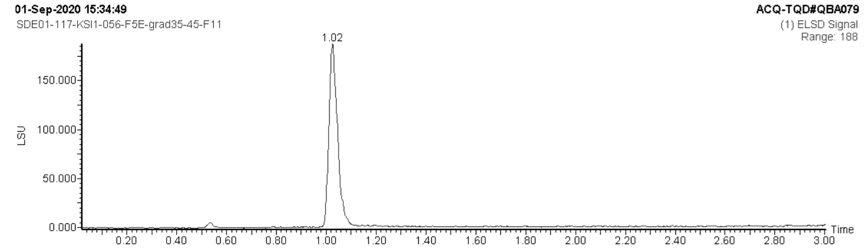

b)

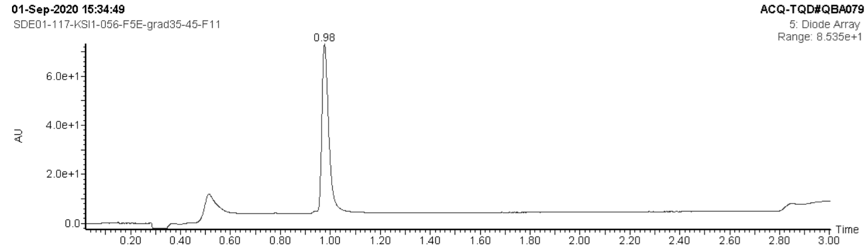

c)

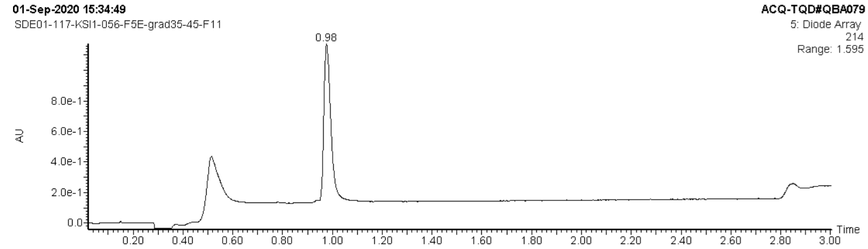

d)

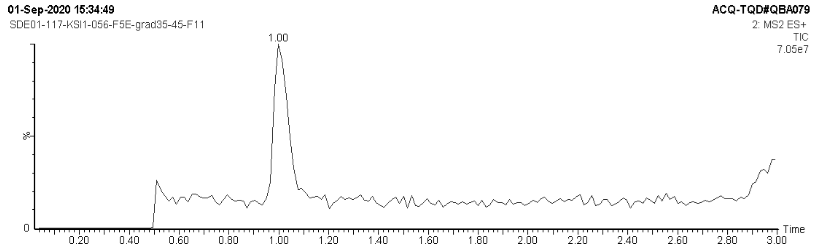

e)

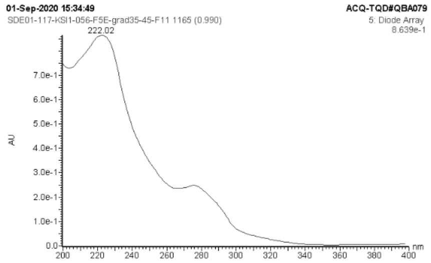

f)

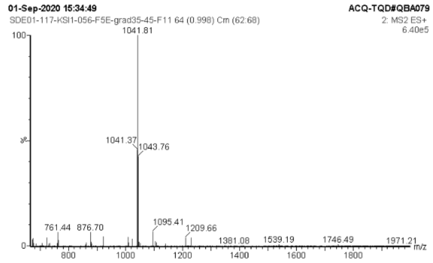

g)

Time (min)	Flow Rate (mL/min)	%A	%B	Curve
Initial	0.600	95.0	5.0	Initial
0.10	0.600	95.0	5.0	6
0.20	0.600	65.0	35.0	6
2.50	0.600	55.0	45.0	6
2.55	0.600	0.0	100.0	6
3.0	0.600	0.0	100.0	6
3.10	0.600	95.0	5.0	6
4.00	0.600	95.0	5.0	6

Figure S15. UPLC analysis of **F_{5E}**. a) ELSD detector, b) Photodiode array detector (absorbance maximum for each compound between 220–500), c) Photodiode array detector (λ:214nm), d) Mass detector (ESI^{+}), e) absorption spectra of the compound, f) Mass spectra of the compound, g) gradient.

Sample name: **F_{7E}** | Solvent A: H_2O+0.1%HCOOH
Column: BEH C18 2.1x50 mm 1.7 µm | Solvent B: Acetonitrile +0,1% HCOOH

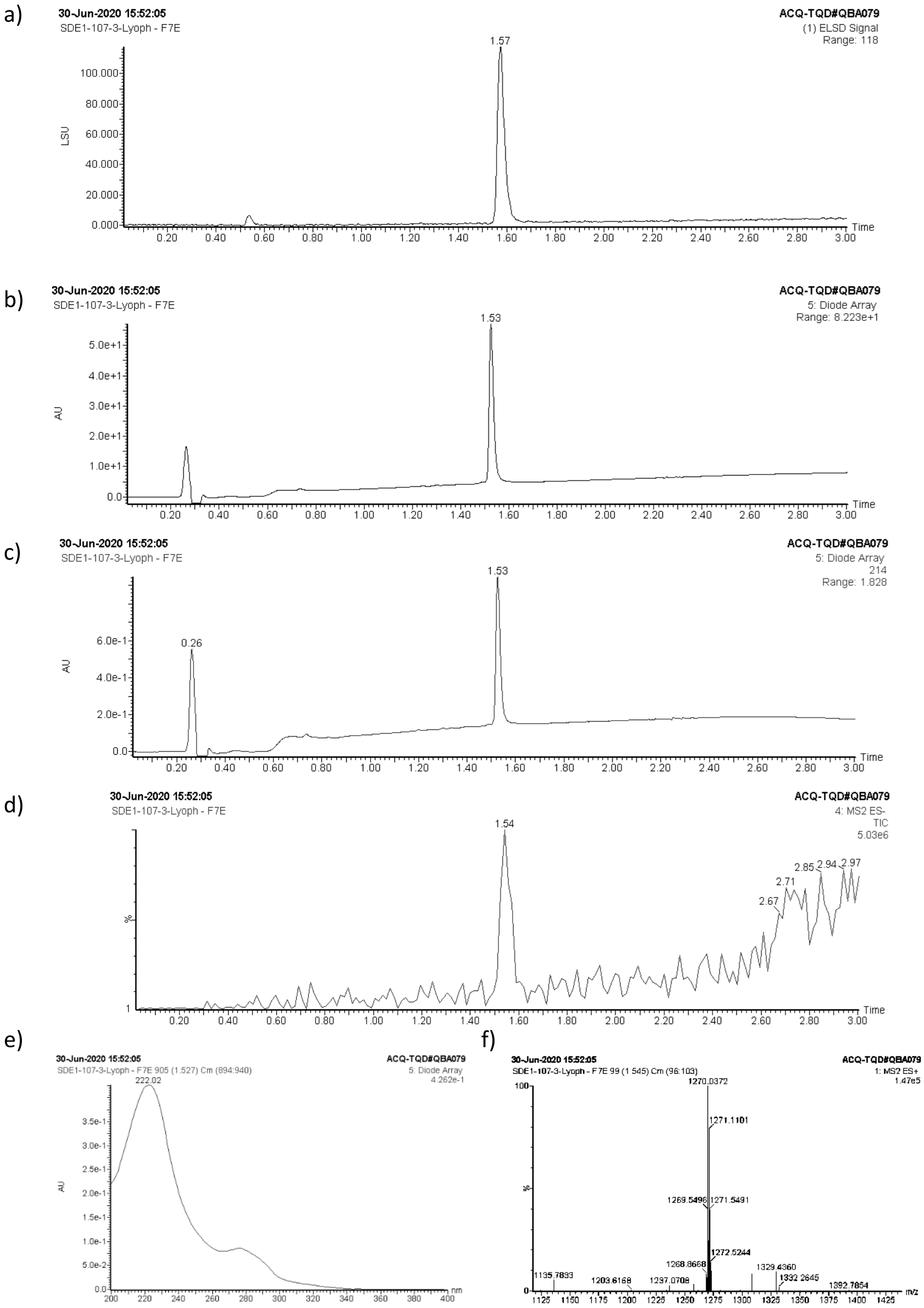

g)

Time (min)	Flow Rate (mL/min)	%A	%B	Curve
Initial	0.600	95.0	5.0	Initial
0.20	0.600	95.0	5.0	6
2.50	0.600	0.0	100.0	6
3.00	0.600	0.0	100.0	6
3.10	0.600	95.0	5.0	6
4.00	0.600	95.0	5.0	6

Figure S16. UPLC analysis of **F_{7E}**. a) ELSD detector, b) Photodiode array detector (absorbance maximum for each compound between 220–500), c) Photodiode array detector (λ:214nm), d) Mass detector (ESI$^+$), e) absorption spectra of the compound, f) Mass spectra of the compound, g) gradient.

Sample name: **F_{9E}**
Column: BEH C18 2.1x50 mm 1.7 µm

Solvent A: H_2O+0.1%HCOOH
Solvent B: Acetonitrile +0,1% HCOOH

a)

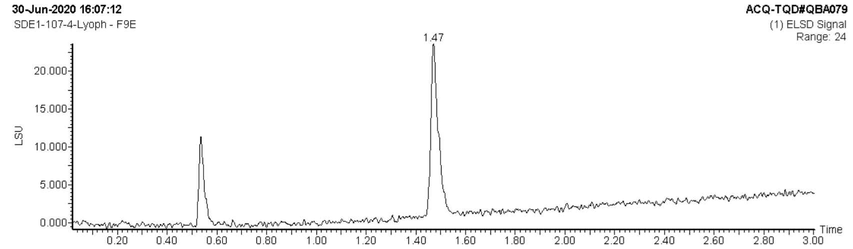

b)

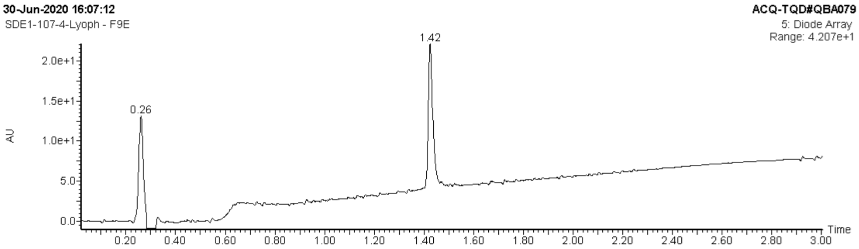

c)

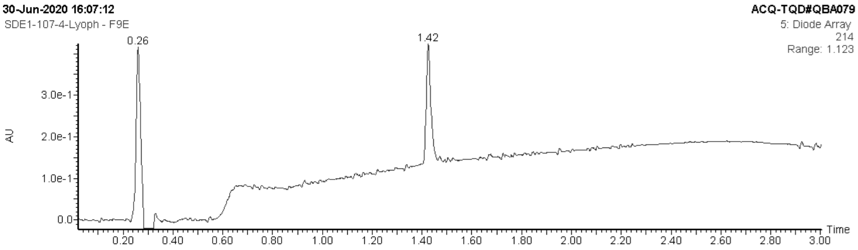

d)

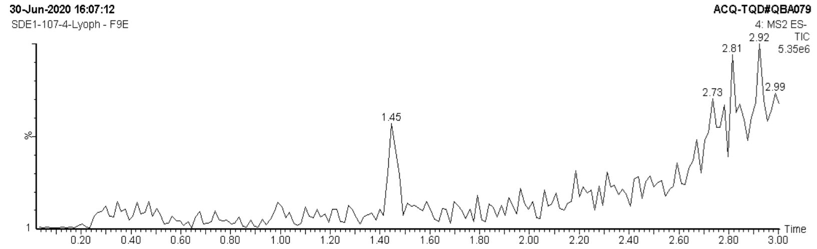

e)

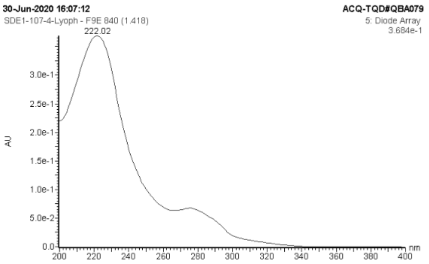

f)

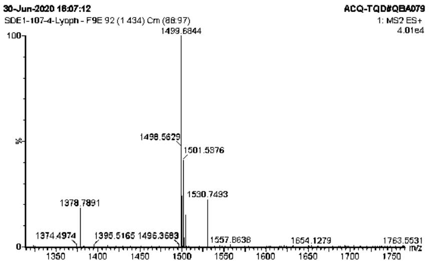

g)

Time (min)	Flow Rate (mL/min)	%A	%B	Curve
Initial	0.600	95.0	5.0	Initial
0.20	0.600	95.0	5.0	6
2.50	0.600	0.0	100.0	6
3.00	0.600	0.0	100.0	6
3.10	0.600	95.0	5.0	6
4.00	0.600	95.0	5.0	6

Figure S17. UPLC analysis of **F_{9E}**. a) ELSD detector, b) Photodiode array detector (absorbance maximum for each compound between 220–500), c) Photodiode array detector (λ:214nm), d) Mass detector (ESI^+), e) absorption spectra of the compound, f) Mass spectra of the compound, g) gradient.

Sample name: **F11E** Solvent A: H_2O+0.1%HCOOH
Column: BEH C18 2.1x50 mm 1.7 µm Solvent B: Acetonitrile +0,1% HCOOH

a)

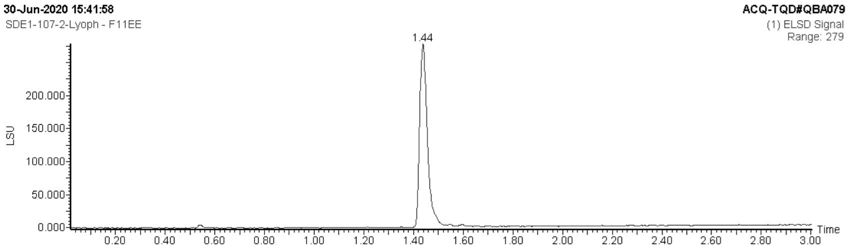

b)

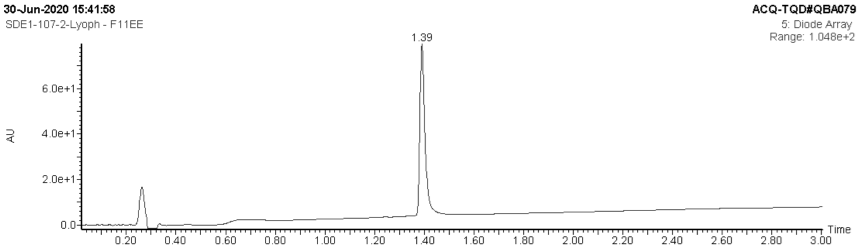

c)

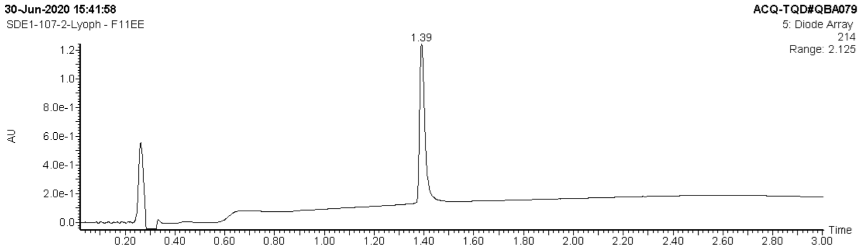

d)

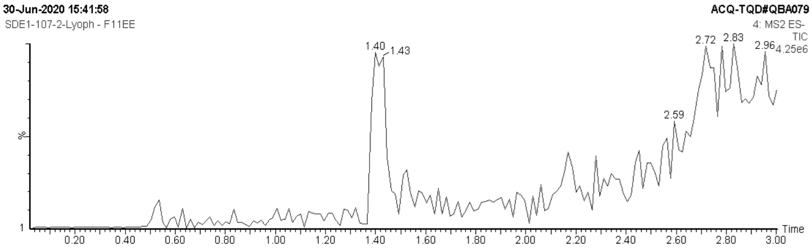

e)

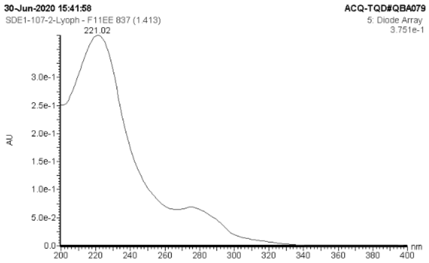

f)

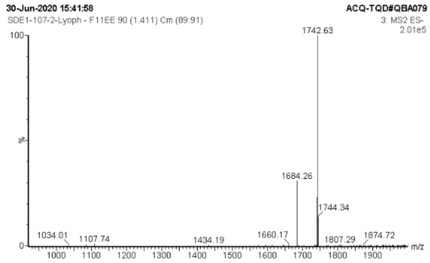

g)

Time (min)	Flow Rate (mL/min)	%A	%B	Curve
Initial	0.600	95.0	5.0	Initial
0.20	0.600	95.0	5.0	6
2.50	0.600	0.0	100.0	6
3.00	0.600	0.0	100.0	6
3.10	0.600	95.0	5.0	6
4.00	0.600	95.0	5.0	6

Figure S18. UPLC analysis of **F11E**. a) ELSD detector, b) Photodiode array detector (absorbance maximum for each compound between 220–500), c) Photodiode array detector (λ:214nm), d) Mass detector (ESI^-), e) absorption spectra of the compound, f) Mass spectra of the compound, g) gradient.

Sample name: **F11SE** Solvent A: H_2O+0.1%HCOOH
Column: BEH C18 2.1x50 mm 1.7 µm Solvent B: Acetonitrile +0,1% HCOOH

a)

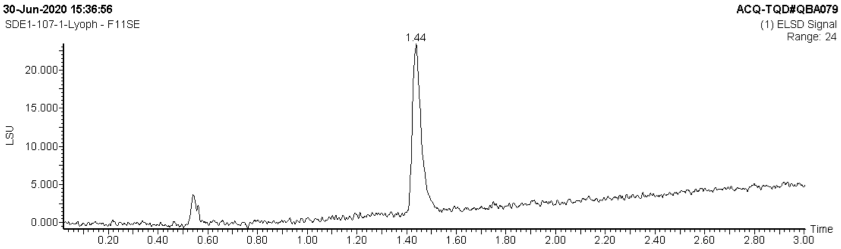

b)

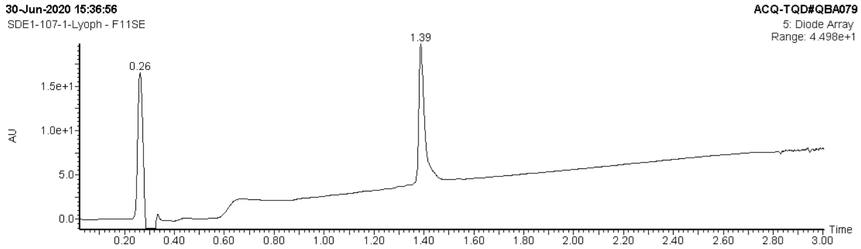

c)

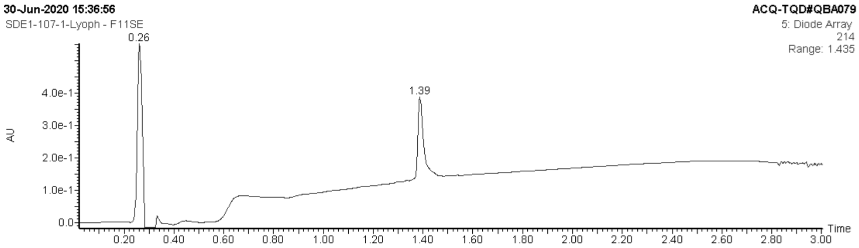

d)

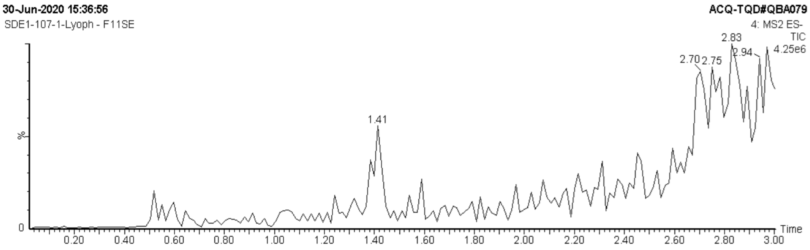

e)

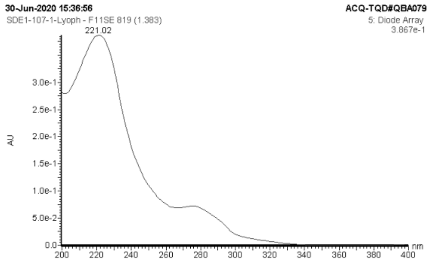

f)

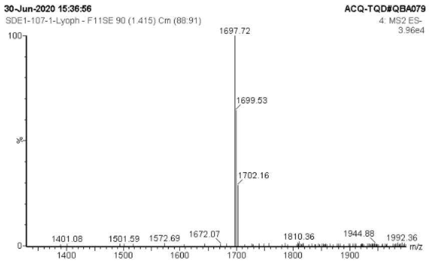

g)

Time (min)	Flow Rate (mL/min)	%A	%B	Curve
Initial	0.600	95.0	5.0	Initial
0.20	0.600	95.0	5.0	6
2.50	0.600	0.0	100.0	6
3.00	0.600	0.0	100.0	6
3.10	0.600	95.0	5.0	6
4.00	0.600	95.0	5.0	6

Figure S19. UPLC analysis of **F11SE**. a) ELSD detector, b) Photodiode array detector (absorbance maximum for each compound between 220–500), c) Photodiode array detector (λ:214nm), d) Mass detector (ESI^-), e) absorption spectra of the compound, f) Mass spectra of the compound, g) gradient.

Sample name: **F11ss** Solvent A: H_2O+0.1%HCOOH
Column: BEH C18 2.1x50 mm 1.7 µm Solvent B: Acetonitrile +0,1% HCOOH

a)

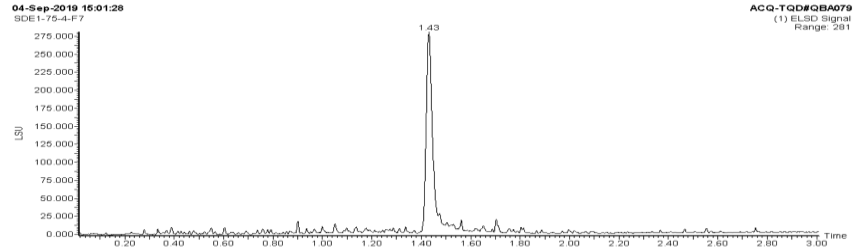

b)

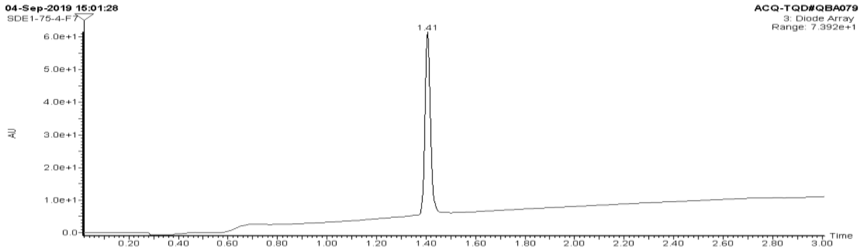

c)

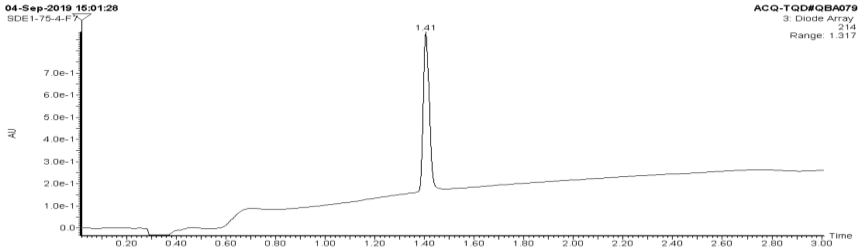

d)

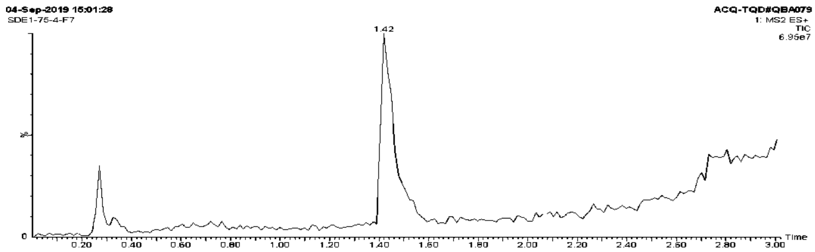

e)

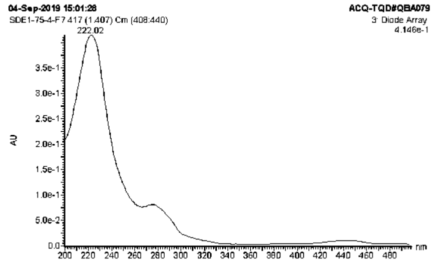

f)

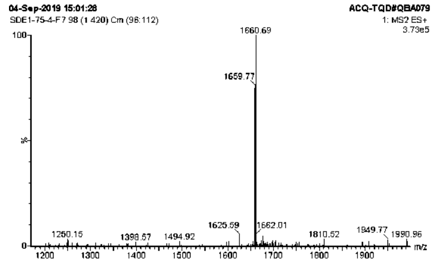

g)

Time (min)	Flow Rate (mL/min)	%A	%B	Curve
Initial	0.600	95.0	5.0	Initial
0.10	0.600	95.0	5.0	6
0.20	0.600	80.0	20.0	6
2.50	0.600	70.0	30.0	6
2.55	0.600	0.0	100.0	6
3.0	0.600	0.0	100.0	6
3.10	0.600	95.0	5.0	6
4.00	0.600	95.0	5.0	6

Figure S20. UPLC analysis of **F11ss**. a) ELSD detector, b) Photodiode array detector (absorbance maximum for each compound between 220–500), c) Photodiode array detector (λ:214nm), d) Mass detector (ESI^+), e) absorption spectra of the compound, f) Mass spectra of the compound, g) gradient.

Sample name: **pF11**	Solvent A: H_2O+0.1%HCOOH
Column: BEH C18 2.1x50 mm 1.7 µm	Solvent B: Acetonitrile +0,1% HCOOH

a)

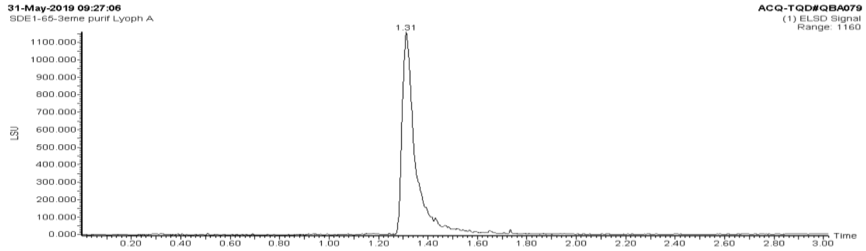

b)

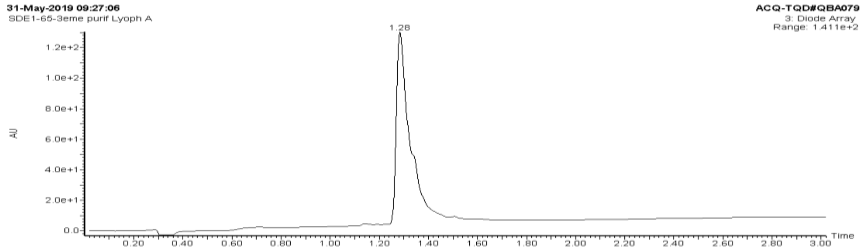

c)

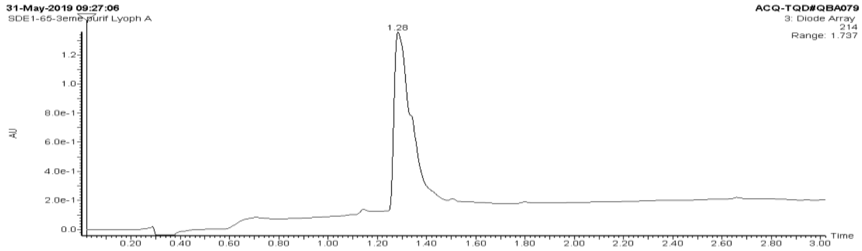

d)

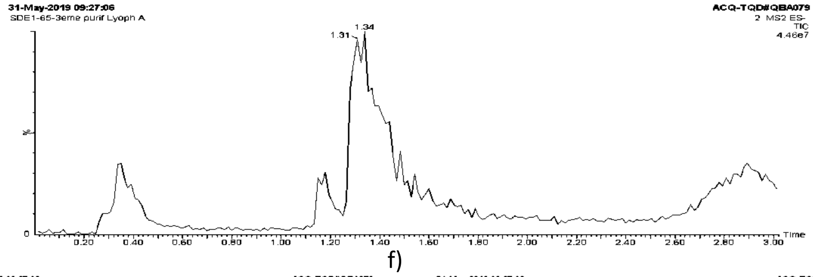

e)

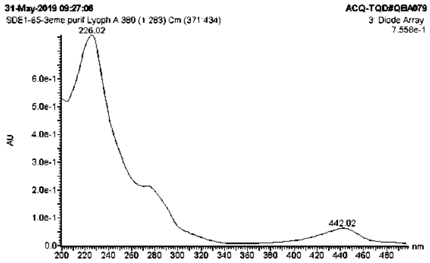

f)

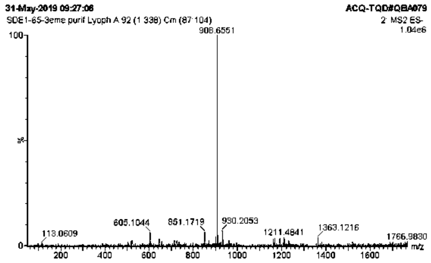

g)

Time (min)	Flow Rate (mL/min)	%A	%B	Curve
Initial	0.600	95.0	5.0	Initial
0.10	0.600	95.0	5.0	6
0.20	0.600	80.0	20.0	6
2.50	0.600	70.0	30.0	6
2.55	0.600	0.0	100.0	6
3.0	0.600	0.0	100.0	6
3.10	0.600	95.0	5.0	6
4.00	0.600	95.0	5.0	6

Figure S21. UPLC analysis of **pF11**. a) ELSD detector, b) Photodiode array detector (absorbance maximum for each compound between 220–500), c) Photodiode array detector (λ:214nm), d) Mass detector (ESI^-), e) absorption spectra of the compound, f) Mass spectra of the compound, g) gradient.

Sample name: **BCN_{5E}**
Column: BEH C18 2.1x50 mm 1.7 µm

Solvent A: H_2O+0.1%HCOOH
Solvent B: Acetonitrile +0,1% HCOOH

a)

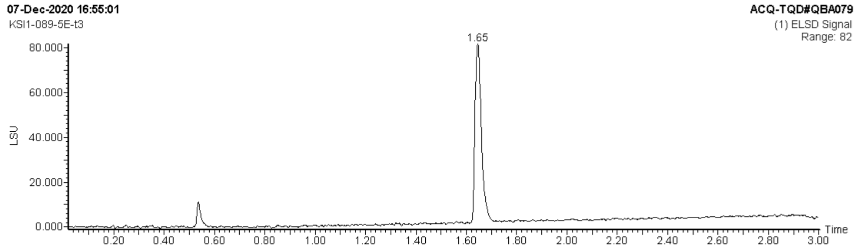

b)

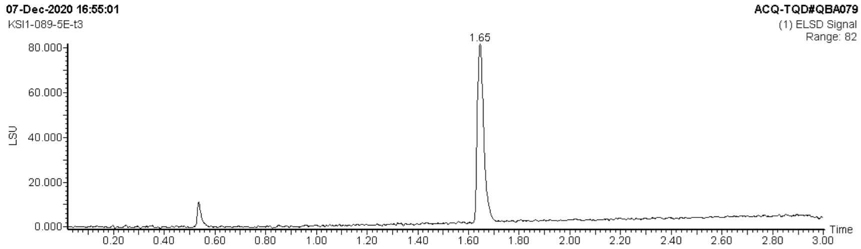

c)

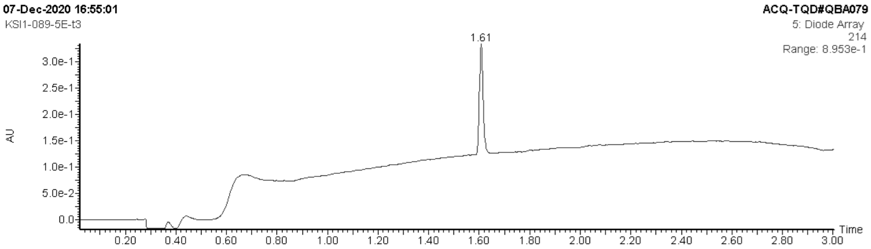

d)

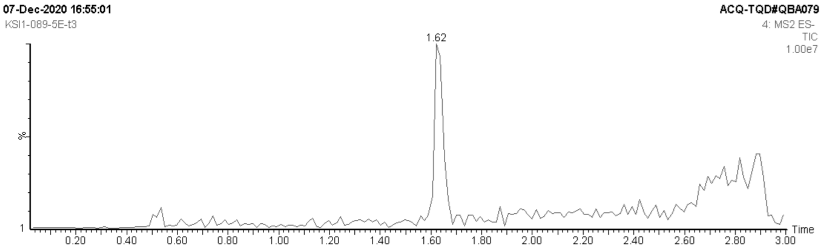

e)

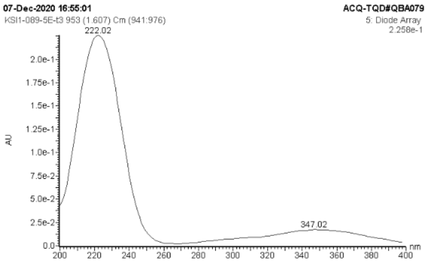

f)

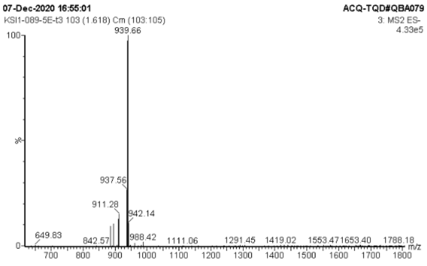

g)

Time (min)	Flow Rate (mL/min)	%A	%B	Curve
Initial	0.600	95.0	5.0	Initial
0.20	0.600	95.0	5.0	6
2.50	0.600	0.0	100.0	6
3.00	0.600	0.0	100.0	6
3.10	0.600	95.0	5.0	6
4.00	0.600	95.0	5.0	6

Figure S22. UPLC analysis of **BCN_{5E}**. a) ELSD detector, b) Photodiode array detector (absorbance maximum for each compound between 220–500), c) Photodiode array detector (λ:214nm), d) Mass detector (ESI^-), e) absorption spectra of the compound, f) Mass spectra of the compound, g) gradient.

Sample name: **BCN$_{6E}$**
Column: BEH C18 2.1x50 mm 1.7 µm

Solvent A: H_2O+0.1%HCOOH
Solvent B: Acetonitrile +0,1% HCOOH

a)

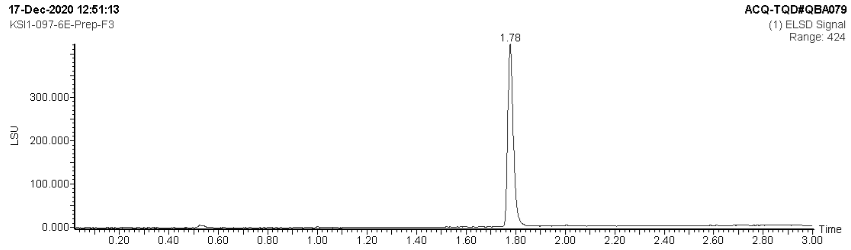

b)

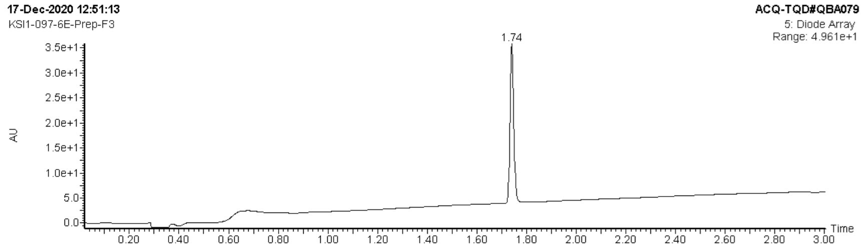

c)

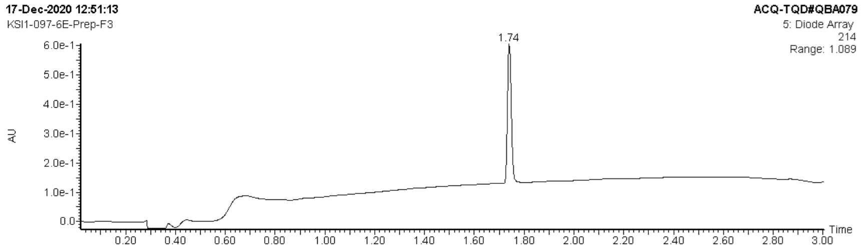

d)

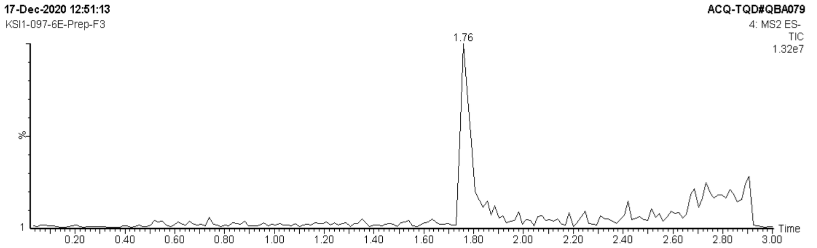

e)

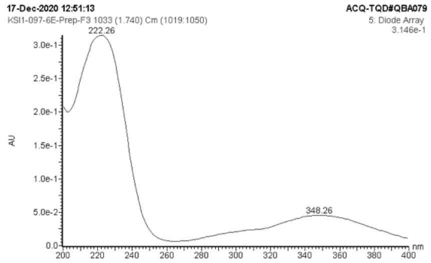

f)

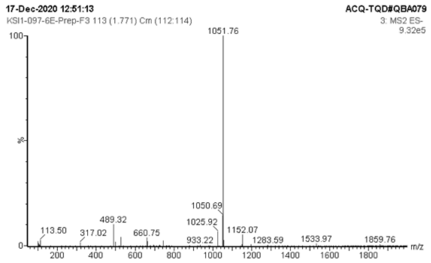

g)

Time (min)	Flow Rate (mL/min)	%A	%B	Curve
Initial	0.600	95.0	5.0	Initial
0.20	0.600	95.0	5.0	6
2.50	0.600	0.0	100.0	6
3.00	0.600	0.0	100.0	6
3.10	0.600	95.0	5.0	6
4.00	0.600	95.0	5.0	6

Figure S23. UPLC analysis of **BCN$_{6E}$**. a) ELSD detector, b) Photodiode array detector (absorbance maximum for each compound between 220–500), c) Photodiode array detector (λ:214nm), d) Mass detector (ESI$^-$), e) absorption spectra of the compound, f) Mass spectra of the compound, g) gradient.

Sample name: **BCN$_{7E}$**
Column: BEH C18 2.1x50 mm 1.7 µm

Solvent A: H_2O+0.1%HCOOH
Solvent B: Acetonitrile +0,1% HCOOH

a)

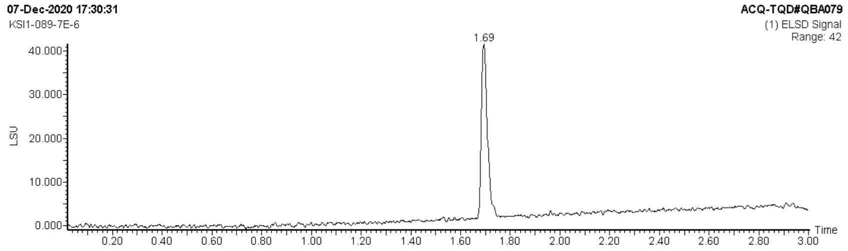

b)

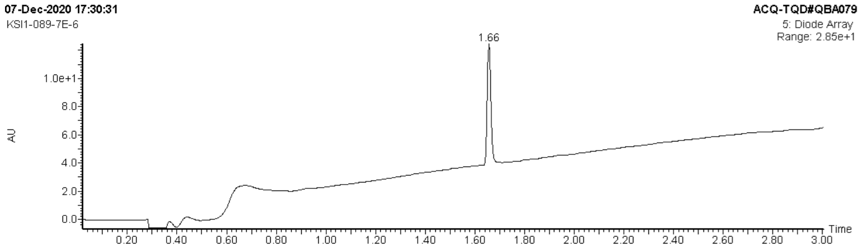

c)

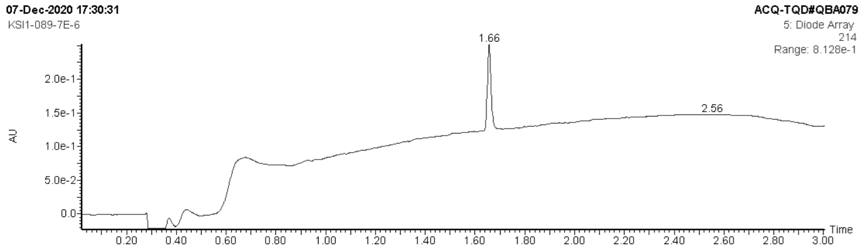

d)

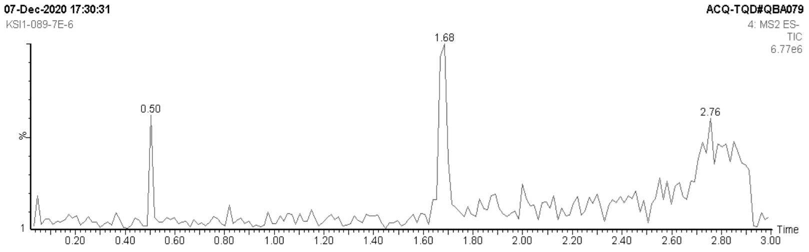

e)

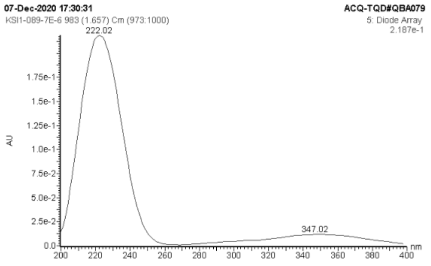

f)

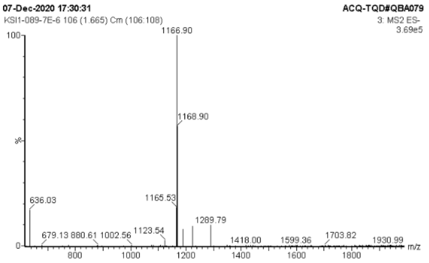

g)

Time (min)	Flow Rate (mL/min)	%A	%B	Curve
Initial	0.600	95.0	5.0	Initial
0.20	0.600	95.0	5.0	6
2.50	0.600	0.0	100.0	6
3.00	0.600	0.0	100.0	6
3.10	0.600	95.0	5.0	6
4.00	0.600	95.0	5.0	6

Figure S24. UPLC analysis of **BCN$_{7E}$**. a) ELSD detector, b) Photodiode array detector (absorbance maximum for each compound between 220–500), c) Photodiode array detector (λ:214nm), d) Mass detector (ESI$^-$), e) absorption spectra of the compound, f) Mass spectra of the compound, g) gradient.

Sample name: **BCN$_{8E}$**
Column: BEH C18 2.1x50 mm 1.7 µm

Solvent A: H_2O+0.1%HCOOH
Solvent B: Acetonitrile +0,1% HCOOH

a)

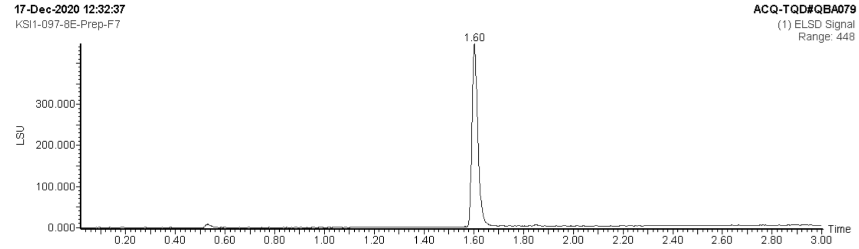

b)

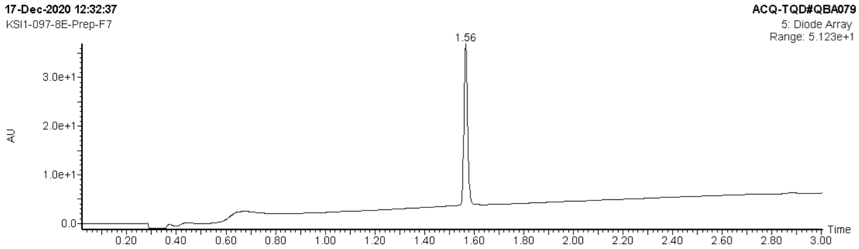

c)

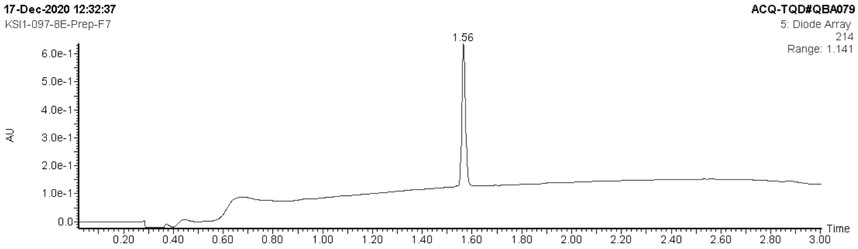

d)

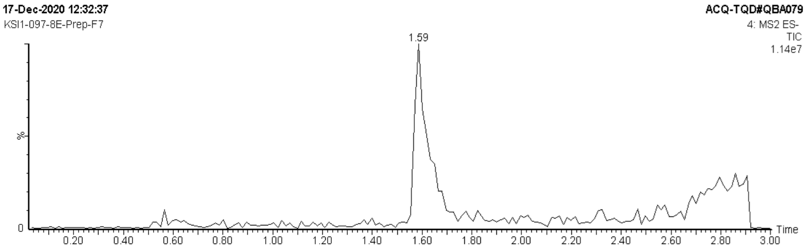

e)

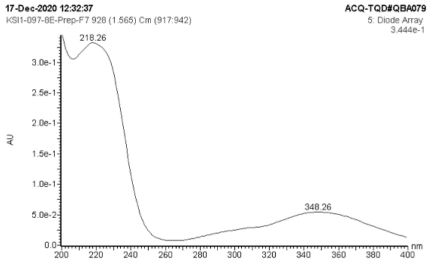

f)

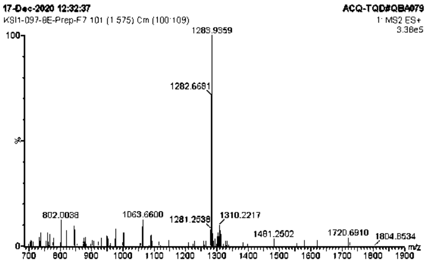

g)

Time (min)	Flow Rate (mL/min)	%A	%B	Curve
Initial	0.600	95.0	5.0	Initial
0.20	0.600	95.0	5.0	6
2.50	0.600	0.0	100.0	6
3.00	0.600	0.0	100.0	6
3.10	0.600	95.0	5.0	6
4.00	0.600	95.0	5.0	6

Figure S25. UPLC analysis of **BCN$_{8E}$**. a) ELSD detector, b) Photodiode array detector (absorbance maximum for each compound between 220–500), c) Photodiode array detector (λ:214nm), d) Mass detector (ESI$^-$), e) absorption spectra of the compound, f) Mass spectra of the compound, g) gradient.

Sample name: **BCN$_{9E}$**
Column: BEH C18 2.1x50 mm 1.7 µm

Solvent A: H_2O+0.1%HCOOH
Solvent B: Acetonitrile +0,1% HCOOH

a)
b)
c)
d)
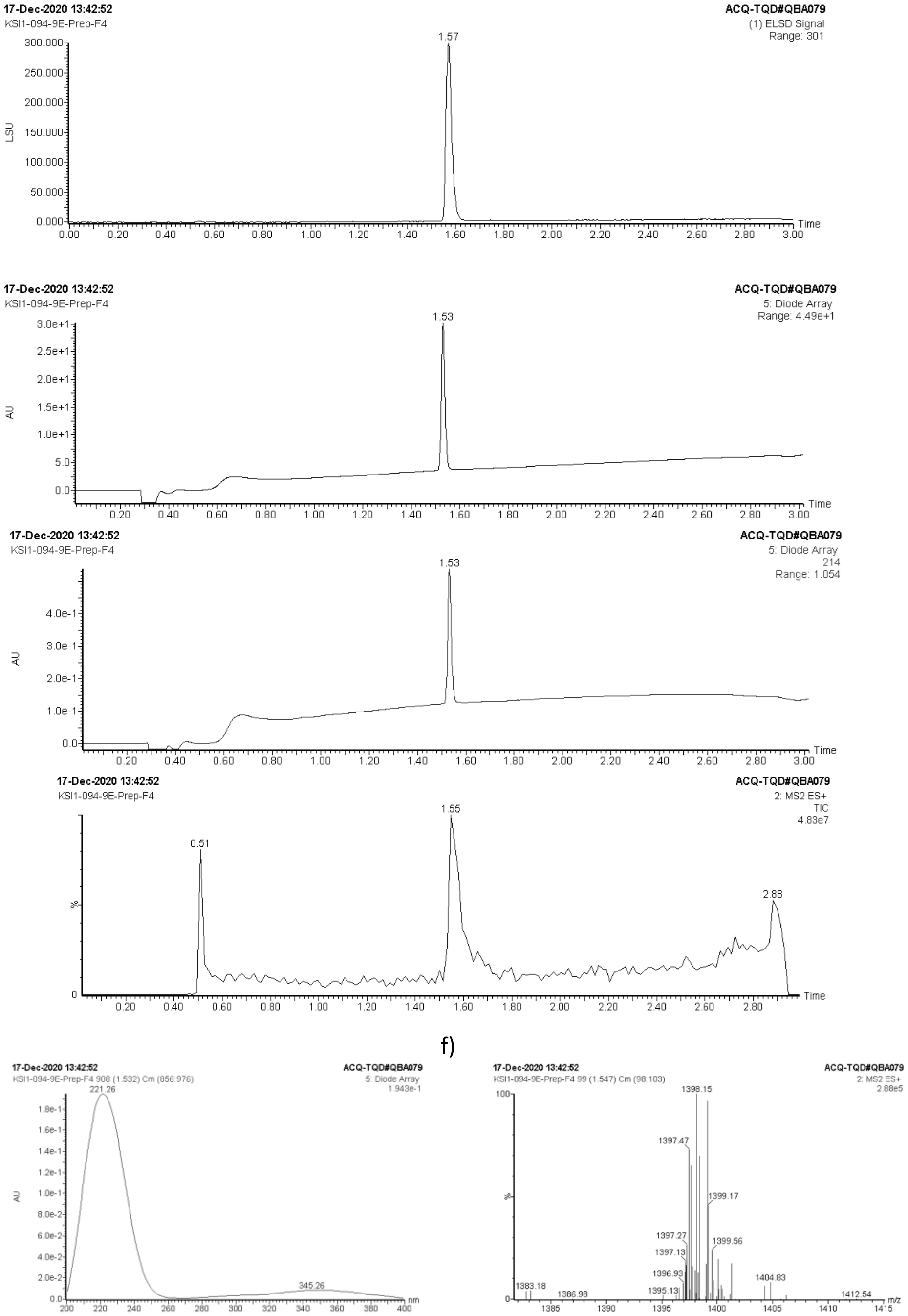

g)

Time (min)	Flow Rate (mL/min)	%A	%B	Curve
Initial	0.600	95.0	5.0	Initial
0.20	0.600	95.0	5.0	6
2.50	0.600	0.0	100.0	6
3.00	0.600	0.0	100.0	6
3.10	0.600	95.0	5.0	6
4.00	0.600	95.0	5.0	6

Figure S26. UPLC analysis of **BCN$_{9E}$**. a) ELSD detector, b) Photodiode array detector (absorbance maximum for each compound between 220–500), c) Photodiode array detector (λ:214nm), d) Mass detector (ESI$^+$), e) absorption spectra of the compound, f) Mass spectra of the compound, g) gradient.

Sample name: **BCN$_{10EE}$** Solvent A: H_2O+0.1%HCOOH

Column: BEH C18 2.1x50 mm 1.7 µm Solvent B: Acetonitrile +0,1% HCOOH

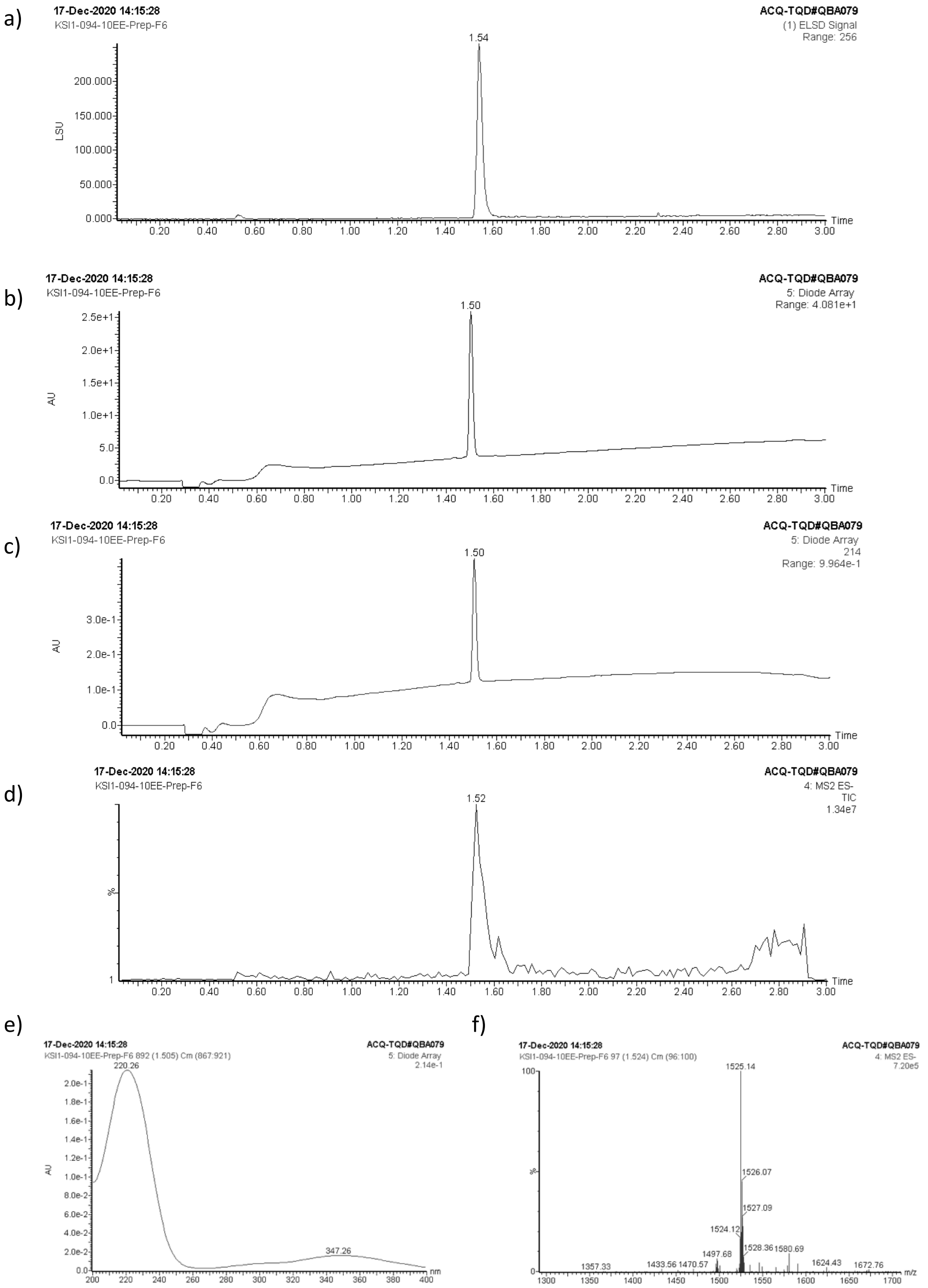

g)

Time (min)	Flow Rate (mL/min)	%A	%B	Curve
Initial	0.600	95.0	5.0	Initial
0.20	0.600	95.0	5.0	6
2.50	0.600	0.0	100.0	6
3.00	0.600	0.0	100.0	6
3.10	0.600	95.0	5.0	6
4.00	0.600	95.0	5.0	6

Figure S27. UPLC analysis of **BCN$_{10EE}$**. a) ELSD detector, b) Photodiode array detector (absorbance maximum for each compound between 220–500), c) Photodiode array detector (λ:214nm), d) Mass detector (ESI$^-$), e) absorption spectra of the compound, f) Mass spectra of the compound, g) gradient.

Sample name: **BCN_{11EE}** Solvent A: H_2O+0.1%HCOOH
Column: BEH C18 2.1x50 mm 1.7 µm Solvent B: Acetonitrile +0,1% HCOOH

a)

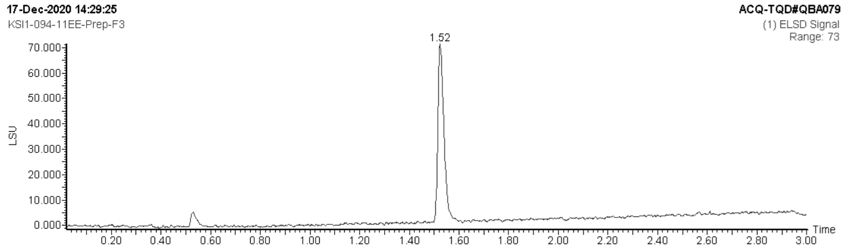

b)

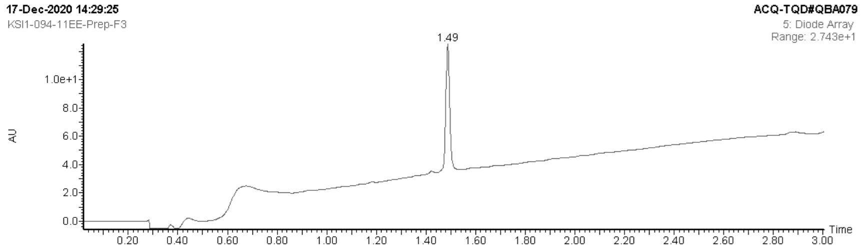

c)

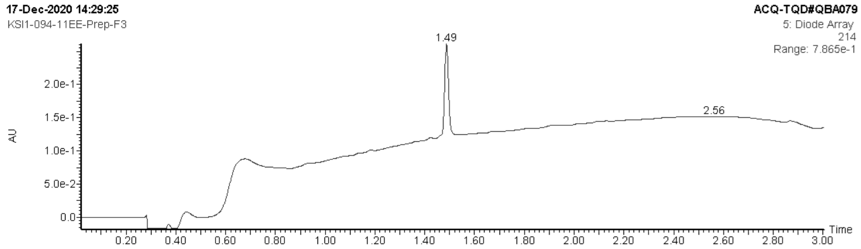

d)

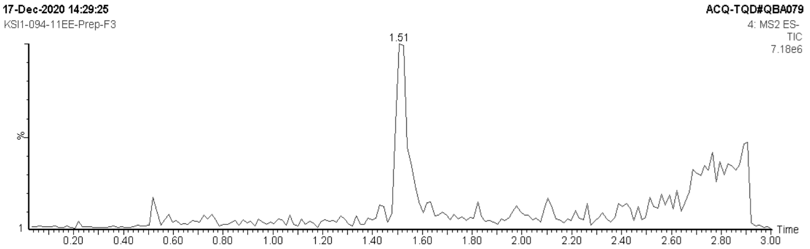

e)

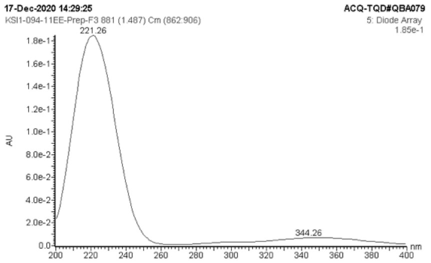

f)

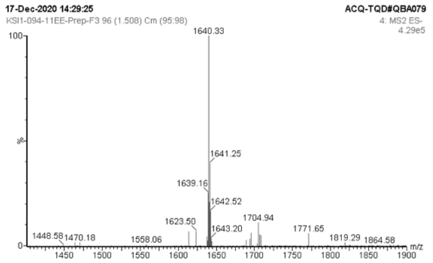

g)

Time (min)	Flow Rate (mL/min)	%A	%B	Curve
Initial	0.600	95.0	5.0	Initial
0.20	0.600	95.0	5.0	6
2.50	0.600	0.0	100.0	6
3.00	0.600	0.0	100.0	6
3.10	0.600	95.0	5.0	6
4.00	0.600	95.0	5.0	6

Figure S28. UPLC analysis of **BCN_{11EE}**. a) ELSD detector, b) Photodiode array detector (absorbance maximum for each compound between 220–500), c) Photodiode array detector (λ:214nm), d) Mass detector (ESI^-), e) absorption spectra of the compound, f) Mass spectra of the compound, g) gradient.

Sample name: **BCN$_{11SE}$** Solvent A: H_2O+0.1%HCOOH
Column: BEH C18 2.1x50 mm 1.7 µm Solvent B: Acetonitrile +0,1% HCOOH

a)

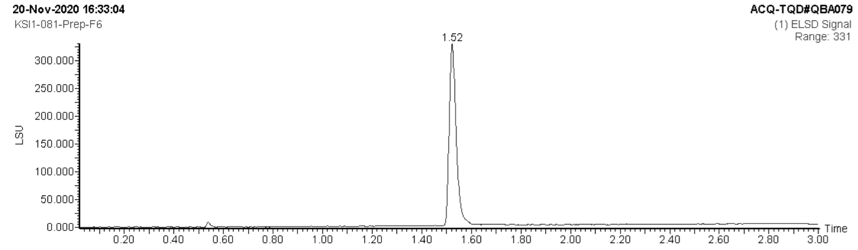

b)

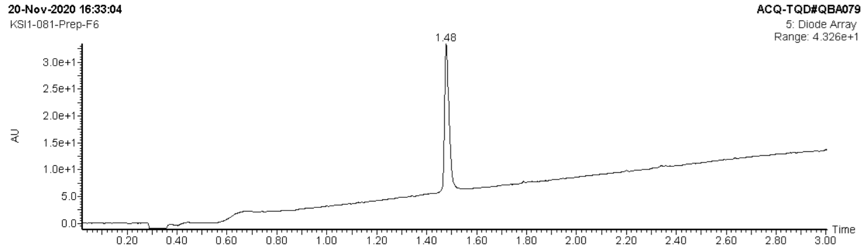

c)

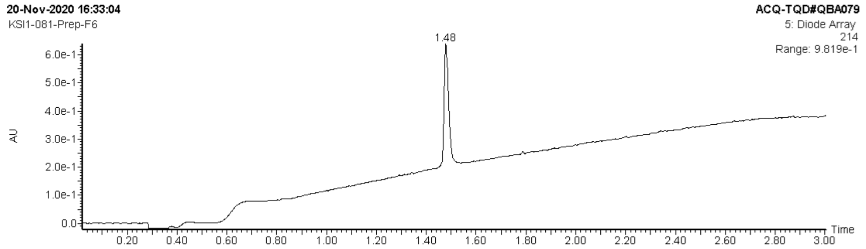

d)

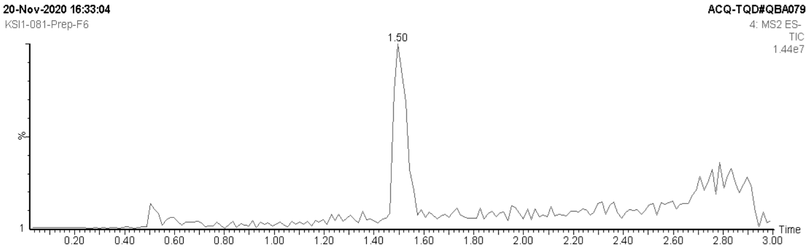

e)

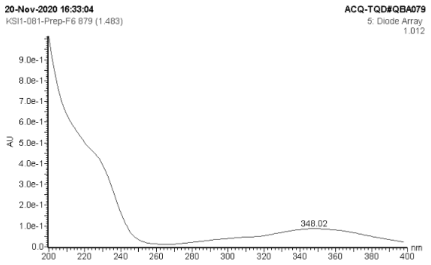

f)

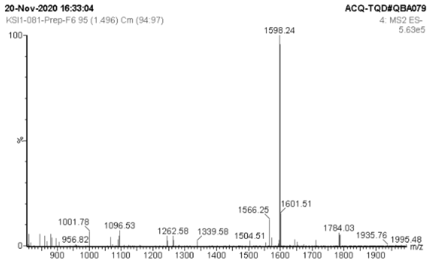

g)

Time (min)	Flow Rate (mL/min)	%A	%B	Curve
Initial	0.600	95.0	5.0	Initial
0.20	0.600	95.0	5.0	6
2.50	0.600	0.0	100.0	6
3.00	0.600	0.0	100.0	6
3.10	0.600	95.0	5.0	6
4.00	0.600	95.0	5.0	6

Figure S29. UPLC analysis of **BCN$_{11SE}$**. a) ELSD detector, b) Photodiode array detector (absorbance maximum for each compound between 220–500), c) Photodiode array detector (λ:214nm), d) Mass detector (ESI^-), e) absorption spectra of the compound, f) Mass spectra of the compound, g) gradient.

Sample name: **Blank**
Column: BEH C18 2.1x50 mm 1.7 µm

Solvent A: H_2O+0.1%HCOOH
Solvent B: Acetonitrile +0,1% HCOOH

a)

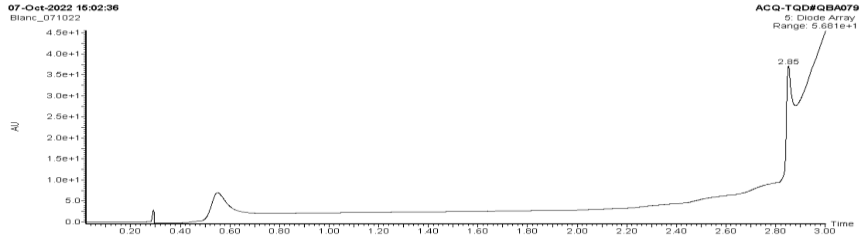

b)

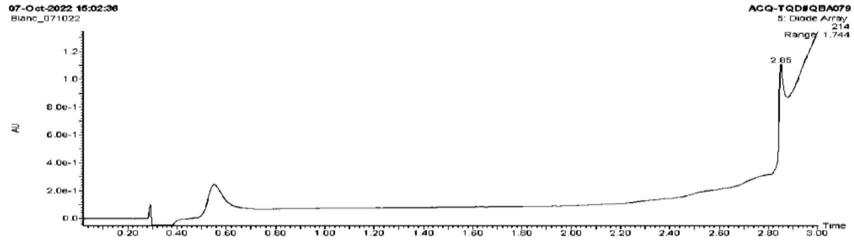

c)

Time (min)	Flow Rate (mL/min)	%A	%B	Curve
Initial	0.600	95.0	5.0	Initial
0.10	0.600	95.0	5.0	6
0.20	0.600	80.0	20.0	6
2.50	0.600	70.0	30.0	6
2.55	0.600	0.0	100.0	6
3.0	0.600	0.0	100.0	6
3.10	0.600	95.0	5.0	6
4.00	0.600	95.0	5.0	6

Figure S30. UPLC analysis of **Blank**. a) Photodiode array detector (absorbance maximum for each compound between 220–500), b) Photodiode array detector (λ:214nm), c) gradient.

HPLC Traces

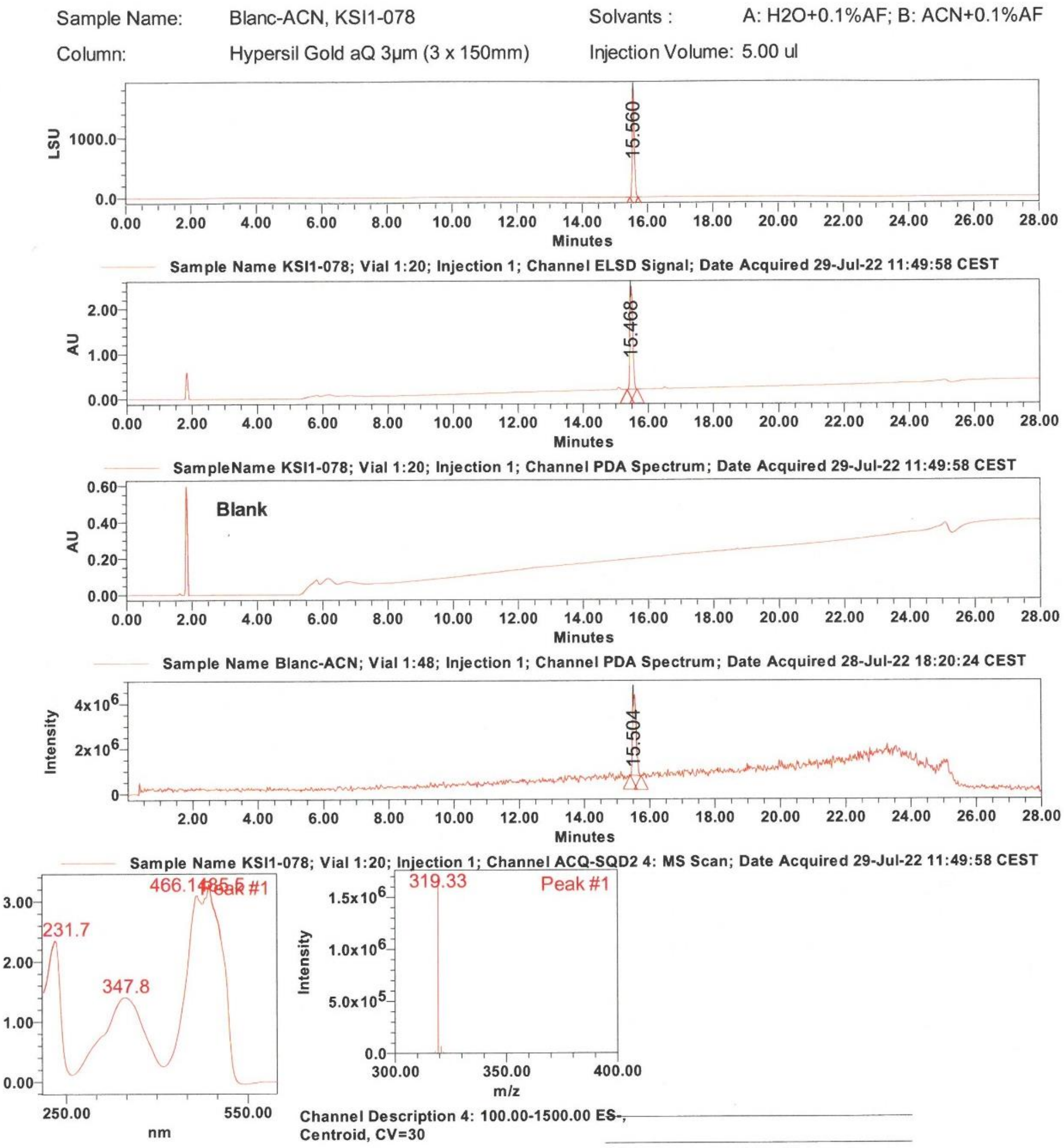

	Time (min)	Flow Rate (mL/min)	%A	%B	%C	%D	Curve
1	Initial	0.500	100.0	0.0	0.0	0.0	Initial
2	3.00	0.500	100.0	0.0	0.0	0.0	6
3	23.00	0.500	0.0	100.0	0.0	0.0	6
4	28.00	0.500	0.0	100.0	0.0	0.0	6
5	29.00	0.500	100.0	0.0	0.0	0.0	6
6	39.00	0.500	100.0	0.0	0.0	0.0	6
7	40.00	0.000	100.0	0.0	0.0	0.0	6

Figure S31. HPLC analysis of **7**. from the top to the bottom: ELSD detector, Photodiode Array Detector, Blank with Photodiode Array Detector and Mass detector (ESI$^-$), absorption spectra of the compound (left), Mass spectra of the compound (right) and gradient.

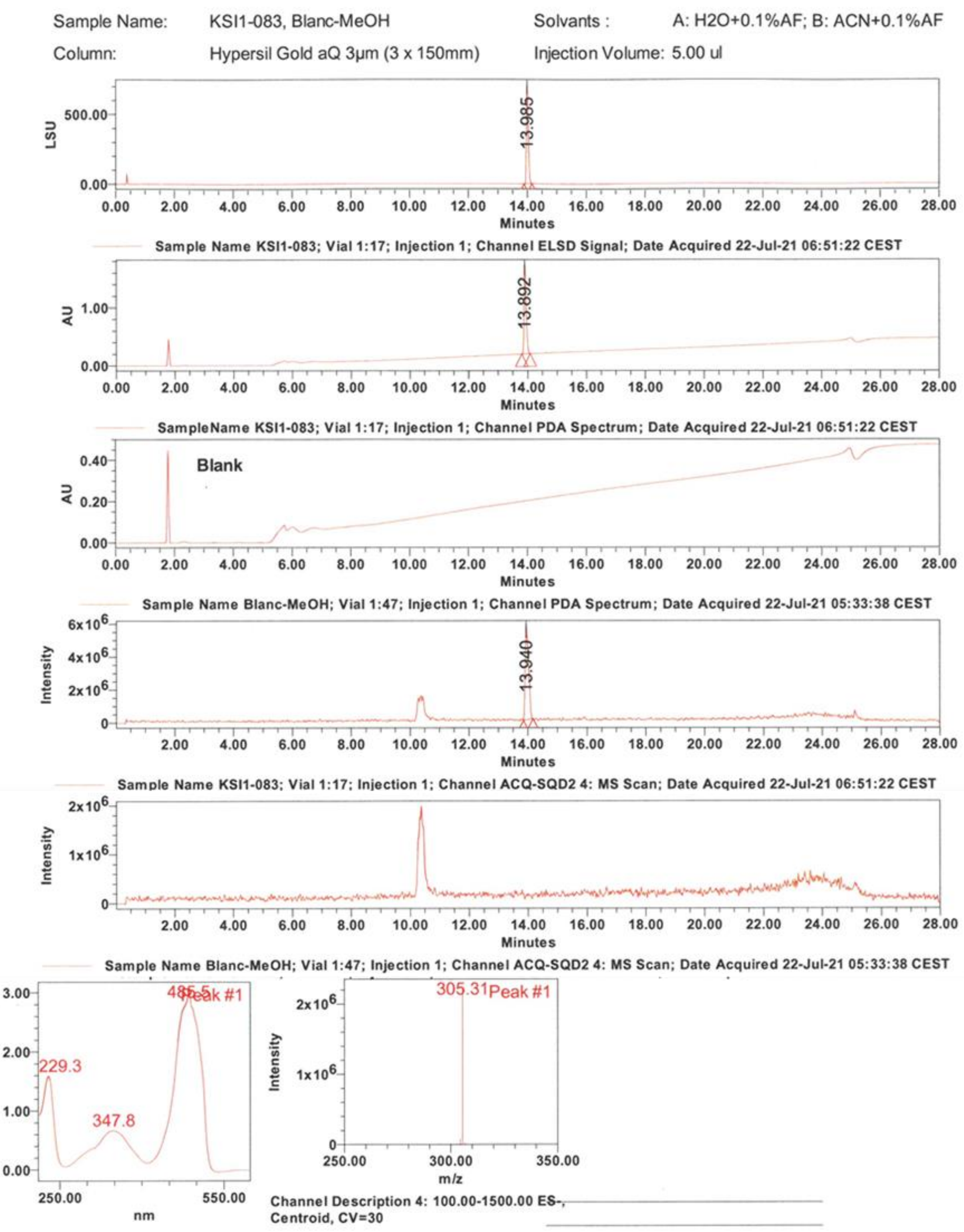

	Time (min)	Flow Rate (mL/min)	%A	%B	%C	%D	Curve
1	Initial	0.500	100.0	0.0	0.0	0.0	Initial
2	3.00	0.500	100.0	0.0	0.0	0.0	6
3	23.00	0.500	0.0	100.0	0.0	0.0	6
4	28.00	0.500	0.0	100.0	0.0	0.0	6
5	29.00	0.500	100.0	0.0	0.0	0.0	6
6	39.00	0.500	100.0	0.0	0.0	0.0	6
7	40.00	0.000	100.0	0.0	0.0	0.0	6

Figure S32. HPLC analysis of **5**. from the top to the bottom: ELSD detector, Photodiode Array Detector, Blank with Photodiode Array Detector, Mass detector (ESI$^-$), Blank with Mass detector (ESI$^-$), absorption spectra of the compound (left), Mass spectra of the compound (right) and gradient.

UV-Visible Absorption and fluorescence Spectra

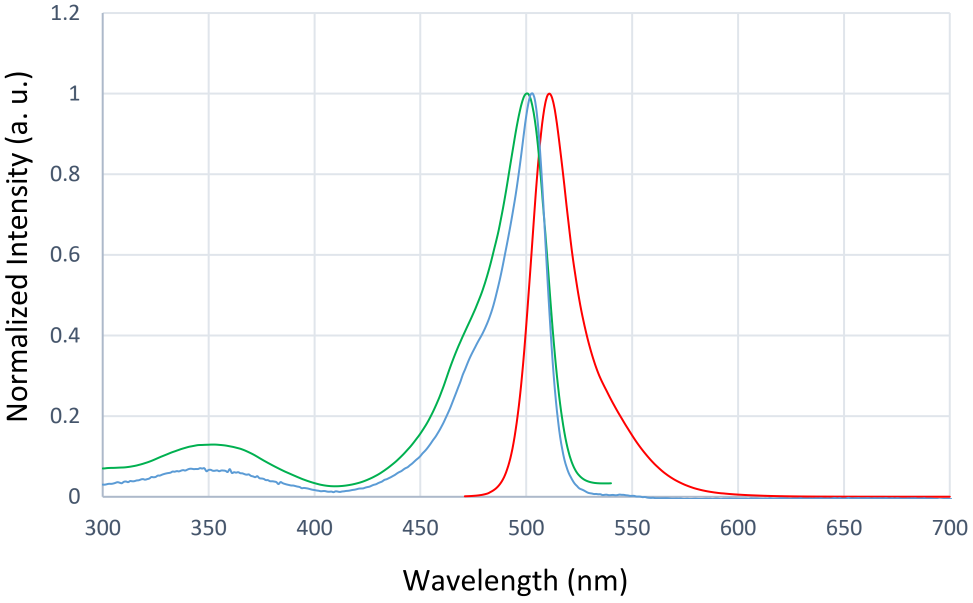

Figure S33. UV-Visible absorption Spectrum (blue) of **BCN_{10EE}** [5.00 µM] in Tris buffer 20mM pH:7.8, recorded on Varian Cary 100 spectrophotometer. Fluorescence excitation (green, λ_{em}= 350nm) and emission spectra (red, λ_{ex} = 450nm) of **BCN_{10EE}** [2.50 µM] in Tris buffer 20mM pH:7.8 at 25°C recorded on HITACI F-2500 spectrofluorometer.
